# Implementing an integrated diabetic foot care programme in Ireland: podiatrists’ experience

**DOI:** 10.1186/s12913-023-10144-z

**Published:** 2023-10-26

**Authors:** Jennifer A. Pallin, Karen Buckley-O’Farrell, Fiona Riordan, Niamh McGrath, Kate O’Neill, Doireann MacLoughlin, Sean F. Dinneen, Claire M. Buckley, Sheena McHugh, Patricia M. Kearney

**Affiliations:** 1 School of Public Health, University College Cork, Cork, Ireland; 2 Alumna of School of Public Health, University College Cork, Cork, Ireland; 3 HRB Evidence Centre, Health Research Board, Grattan House, 67-72 Lower Mount Street, Dublin 2, Ireland; 4 Chronic Disease Management Hub Clare / Limerick, MWCH, Clare, Ireland; 5 School of Medicine, University of Galway, Galway, Ireland; 6 Centre for Diabetes, Endocrinology and Metabolism, Galway University Hospitals, Galway, Ireland

**Keywords:** Diabetic foot, Integrated care, Survey, Model of Care

## Abstract

**Background:**

International evidence suggests that an integrated multidisciplinary approach to diabetic foot management is necessary to prevent ulceration and progression to amputation. Many health systems have introduced policies or models of care supporting the introduction of this evidence into practice, but little is known about the experiences of those involved in implementation. This study addresses this gap by examining the experiences of podiatrists providing integrated diabetic foot care.

**Methods:**

Between October 2017 and April 2018, an online survey comprising closed and open-ended questions on podiatrists’ demographics, clinical activity, links with other services, continuous professional development activities and experiences of implementing the Model of Care was administered to podiatrists (n = 73) working for Ireland’s Health Service Executive in the community and hospital setting. Data were analysed using descriptive statistics and qualitative content analysis.

**Results:**

The response rate was 68% (n = 50), with 46% (n = 23), 38% (n = 19) and 16% (n = 8) working across hospital, community and both settings, respectively. Most reported treating high-risk patients (66%), those with active foot disease (61%) and educating people about the risk of diabetes to the lower limb (80%). Reported challenges towards integrated diabetic foot care include a perceived lack of awareness of the role of podiatry amongst other healthcare professionals, poor integration between hospital and community podiatry services, especially where new services had been developed, and insufficient number of podiatrists to meet service demands.

**Conclusion:**

Previous evidence has shown that there is often a gap between what is set out by a policy and what it looks like when delivered to service users. Results from the current study support this, highlighting that while most podiatrists work in line with national recommendations, there are specific gaps and challenges that need to be addressed to ensure successful policy implementation.

**Supplementary Information:**

The online version contains supplementary material available at 10.1186/s12913-023-10144-z.

## Background

Diabetic foot disease, including ulceration and amputation, is one of the most common lower extremity complications of diabetes, with the lifetime risk of a person with diabetes developing a diabetic foot ulcer estimated to be as high as 34% [1]. Diabetic foot ulcers increase a person’s risk of amputation and are associated with increased health-related and financial burden to patients, society, and health systems [1–5].

However, diabetic foot ulcers and subsequent amputations can be prevented through timely identification of risk factors and referral to appropriate healthcare professionals in primary, secondary, and tertiary care settings [6]. This requires involvement of several clinicians across different care settings and institutions [6, 7]. Translating research evidence into policy is crucial to improving the evidence base of health care as well as to improving health care outcomes [8]. As a result of this need, internationally, models of integrated care have become a pillar for management of diabetes, and associated complications, to address service fragmentation, improve patient experience, and achieve better efficiency and value from healthcare systems [9–13]. 

In Ireland, as part of a reform programme orientated towards delivery of integrated diabetes care, a Model of Care for the Diabetic Foot was developed and published in 2011 by Ireland’s National Clinical Programme for Diabetes (NCPD) [14]. This model of care was adapted from the National Institute for Health and Care Excellence (NICE) guidelines, and sought to standardise and improve diabetic foot care by reorganising existing, fragmented services, and outlining *what* care people with diabetes should receive and *where* and *by whom* their care should be delivered depending on their level of risk for ulceration and amputation.

The outlined pathway of care is similar to those in other health systems [14–17]. It provides recommendations on a diabetic foot care pathway based on risk categories, with those at low risk for ulceration (i.e., having no risk factors) remaining under the care of their GP, those at moderate risk (≤ 1 risk factor) or high risk (≥ 2 risk factors) being referred to hospital or community podiatry for annual review and care as needed, and those with active foot disease being actively managed by a multidisciplinary specialist foot care service in the hospital setting, in conjunction with vascular surgery, orthopaedics and orthotics input as required [14].

However, evidence suggests there is often a disconnect between what is set out in models of care or policies and what is adopted by services and healthcare professionals [18, 19]. In addition, we know that implementation often varies across contexts, which may potentially limit their impact on population health [7, 19]. Specific to diabetic foot care, reported success of these models of integrated care has been inconsistent. Some have shown these models to increase understanding of the impact of diabetes on the lower limb among healthcare professionals involved in diabetes care, increase use of appropriate referral pathways and lead to reductions in lower extremity amputations [9, 10]. Whereas others report a lack of coordination and communication among clinicians within and between organisations and healthcare settings [7, 13, 18]. Yet, little is known about the specific experiences of personnel involved in implementing an integrated diabetic foot care service. In addition, little is known about what supports they need to enable successful policy implementation. This is despite the recognised need for understanding personnel and organisational experiences of implementing policies so relevant stakeholders can better understand both the impact of the programme, how they work and for whom, and what is needed to sustain them in the real-world setting [8, 13, 18].

With podiatrists being recognised as a key component of integrated diabetic footcare delivery, their experiences, and perceptions of implementing such programmes is critical towards identifying areas of good practice and enabling implementation of policy into practice [20, 21]. In Ireland, recent studies have shown that in the hospital setting, podiatrists are often unable carry out activities as outlined within the Model of Care due to service demand. In addition, they were not always supported by allied healthcare professionals when it came to developing the podiatry service [13]. Similar findings have been reported internationally [7, 22]. However, there is still a dearth of information on podiatrists’ specific experiences and whether there were specific processes that promoted or inhibited implementation of this diabetic foot model of care into practice. Therefore, this study explores whether podiatrists’ work activities align with recommendations within the Model of Care, and their experiences of providing diabetic foot care since its introduction with the aim of identifying barriers to service delivery and areas for improvement. Results will inform stakeholders who are involved in decision making around integrated diabetic foot care programmes internationally on what factors need to be considered to enable successful policy implementation.

## Research design and methods

### Study setting

Ireland’s Model of Care for the Diabetic Foot recommends that diabetic foot care be delivered across hospital, community, and primary care services with the level of care depending on the patient’s risk for ulceration and amputation [14]. Hospitals are organised into seven groups, with each group consisting of smaller local hospitals (referred to as Model 2 or 3 hospitals) and large hospitals within urban centres (referred to has Model 4 hospitals) that cater for highly specialised and complex care. Primary care and community services are provided through nine community healthcare organisations (CHOs). Each hospital group and their respective CHO (See Figure S1 and Table S1 for an overview of hospital groups and CHOs) work together to support integrated care [23]. Podiatrists working within Ireland’s Health Service Executive (HSE) are based within community and/or hospital settings. Depending on their level of experience and expertise, podiatrists can be employed as staff grade, senior, clinical specialists, and clinical podiatry manager. Those employed as a basic grade typically have ≤ 3 years’ experience working as a podiatrist since graduating from university, and those employed as a senior typcially have ≥ 3 years’ experience. Those employed as clinical specialist must demonstrate evidence of continuing professional development, in the form of post-graduate qualifications relevant to a specialist diabetes foot service and are responsible for co-ordination of strategic planning and development of specialist diabetice foot services. Finally, podiatry managers are responsible for the development and delivery of high-quality podiatry services across their respective CHO. They also contribute to strategic development in conjunction with allied healthcare professionals and partake with other management teams in fostering cross divisional integrated working. The current study explores podiatrists’ experience of providing diabetic foot care seven years after implementation of the first Model of Care for the diabetic foot (2011).

### Survey development

Survey development (See supplementary information for survey) was informed by multiple sources, including Ireland’s Model of Care for the diabetic foot and a previously published survey in the United Kingdom developed to examine provision of, and variation in, hospital-based diabetes services in the United Kingdom [24]. The survey was modified for the current study to explore service provision in community and hospital-based diabetes podiatry services across Ireland. The NCPD national clinical lead for podiatry (DM) was also consulted during questionnaire development to ensure it reflected podiatry work settings and practices in Ireland. According to them, many podiatrists work in multiple settings (e.g., community, hospital, private practice), providing a combination of diabetic foot and non-diabetic foot care, and so similar questions were asked but response options were tailored depending on podiatrist’s work setting. Those working in both hospital and community settings, were provided all possible responses for both settings. It was piloted with two podiatrists working in hospital and community settings.

### Survey content

The survey comprised closed ended questions addressing podiatrists’ demographics, clinical activity, links with other services, satisfaction with hospital and community services, and continuous professional development (CPD) activities. Where participants indicated they did not carry out specific clinical activities, they were asked to explain why in an open-ended question box. Depending on work setting, respondents were also asked to indicate their satisfaction with hospital and community services using a 6-point Likert scale (i.e., 1 = very satisfied, 2 = satisfied, 3 = neither satisfied nor unsatisfied, 4 = unsatisfied, 5 = very unsatisfied 6 = I don’t know if this happens).

One open-ended question at the end of the survey asked *“We value your insight into diabetes care. Please use the space provided to describe your experiences of implementing the National Model of Diabetic Foot Care. If applicable, what are the changes you would make to the National Clinical Programme for Diabetes?”.*

### Population and recruitment procedures

All podiatrists working within the HSE were eligible for inclusion. The NCPD compiled a list of 73 podiatrists working within the HSE and the NCPD national clinical lead for podiatry distributed the survey, online via SurveyMonkey, on behalf of the research team. Although all podiatrists worked within the HSE, some were employed by voluntary organisations. It was also distributed by Ireland’s three professional associations for podiatrists and chiropodists to their members (i.e., the the Society of Chiropodists and Podiatrists of Ireland, the Institute of Chiropodists and Podiatrists (Irish branch) and the Irish Chiropodists / Podiatrists Organization Ltd). Participants received an initial recruitment email on 31st October 2017, and reminders on the 17th November and 8th December 2017. A final reminder email was sent on 6th April 2018. The survey was closed on 20th April 2018. The opening page of the survey contained a study information sheet and consent form. Once participants selected the consent box they were directed to the survey. It was not possible to begin the survey until consent was confirmed.

### Data analysis

Only data relating to their work practices within the HSE are reported here, as this study aims to explore the experience of those working within Ireland’s public health system.

### Quantitative data analysis

Data were downloaded and cleaned in Excel before importing into Stata/BE 17. Descriptive statistics (means, percentages) were generated for participant demographics, workplace activities carried out, referral access to other members of the multidisciplinary team and satisfaction with hospital and community management of the at-risk foot in diabetes. Respondents were asked to state the community health organisation (CHO) where they worked, and the number of podiatrists per 100,000 in each CHO was calculated from the population in that CHO [23]. Variables indicating podiatrists’ satisfaction with acute and community services were collapsed from a 6-point Likert scale to a 4-point Likert scale (i.e., very satisfied, and satisfied collapsed into satisfied and very dissatisfied and dissatisfied into dissatisfied).

### Qualitative data analysis

The online software tool NVivo version 12 (www.qsrinternational.com) was used for data analysis of the open-ended question at the end of the survey. Data were analysed using content analysis according to Hsieh & Shannon which they describe as “a research method for the subjective interpretation of the content of text data through the systematic classification process of coding and identifying themes or patterns” [25]. This method was selected as it would allow for systematic coding and categorizing of responses, and examination of core themes. We applied an inductive data-driven approach as it allowed for examination of core themes for a phenomenon with limited existing theory or research literature. First, responses to the open-ended question, and information relating to setting and number of years of service, were compiled into one Microsoft Word document and read by one researcher (JP) to familiarise themselves with the data. Second, all data was imported into NVivo. Third, data was coded into superordinate themes, including positive and negative experiences and changes they would make to the NCPD or Model of Care. Fourth, working through the text line-by-line data was coded into sub-categories to identify what led to these experiences and whether there were specific changes podiatrists would make. Finally, we conducted frequency analysis of the categories and sub-categories to explore whether certain challenges were experienced more frequent than others.

## Results

### Quantitative

#### Demographic characteristics

The response rate was 68.5% (n = 50), with 46% (n = 23) working within the hospital setting only (H), 38% (n = 19) working in the community setting only (C) and 16% (n = 8) working across both settings (HC). In addition, 24% (n = 12) also engaged in private practice. Although all worked within the HSE, some were employed by voluntary organisations (see Table 1). Respondents had been working as a podiatrist for mean (SD) 13.54 (± 1.4) years, providing diabetic foot care for 12.36 (± 1.2) years and working in their current post for 6.46 (± 0.95) years. For those working in the hospital setting, most of their patients had a diagnosis of diabetes, with this patient group accounting for mean (SD) 91.6% (5.9) of their client load. Similar results were seen in the community setting, with people with diabetes accounting for 72% (7.1) of their client load. Regarding hospital-based podiatrists (n = 31), 13% (n = 4), 29% (n = 9) and 45% (n = 14) worked within a Model 2, Model 3, and Model 4 hospital, respectively. Table 1 also outlines the number of podiatrists by CHO and the calculated number of podiatrists working within each CHO per 100,000 population.

**Table 1:** Respondent demographics

	Hospital (n = 23) N (%)	Community (n = 19) N (%)	Hospital & Community (n = 8) N (%)
**Age (Years)** *21–30* *31–40* *41–50* *51–60*	7 (30.4) 8 (34.8) 7 (30.4) 1 (4.4)	7 (36.8) 4 (21.1) 5 (26.3) 3 (15.8)	1 (12.5) 1 (12.5) 4 (50) 2 (25)
**Education level** *Higher diploma* *Undergraduate* *Postgraduate* *Missing*	0 14 (60.8) 8 (34.8) 1 (4.4)	1 (5.3) 14 (73.7) 4 (21) 0	0 5 (62.5) 2 (25) 1 (12.5)
**Employer** *HSE* *Diabetes Ireland* *Voluntary hospital* *Missing*	18 (79) 0 4 (17) 1 (4)	9 (47.3) 4 (21) 0 6 (31.6)	7 (87.5) 1 (12.5) 0 0
**Job title** *Staff grade* *Senior* *Clinical Specialist* *Manager*	3 (13) 16 (69.6) 4 (17.4) 0	2 (10.5) 15 (79) 0 2 (10.5)	0 7 (87.5) 0 1 (12.5)
**Community health organisation (CHO)** *CHO 1* *CHO 2* *CHO 3* *CHO 4* *CHO 5* *CHO 6* *CHO 7* *CHO 8* *CHO 9* *Missing*	1 (4.4) 1 (4.4) 1 (4.4) 4 (17.6) 3 (13.2) 1 (4.4) 1 (4.4) 2 (8.8) 8 (35.2) 1 (4.4)	5 (26.3) 0 3 (15.8) 5 (25.3) 2 (10.6) 0 0 0 3 (15.8) 1 (5.3)	1 (12.5) 2 (25) 1 (12.5) 2 (25) 1 (12.5) 0 0 0 1 (12.5) 0
**Community health organisation (total population for that CHO)** ^$^ *CHO 1 (389,048)* *CHO 2 (445,356)* *CHO 3 (379,327)* *CHO 4 (664,533)* *CHO 5 (497,578)* *CHO 6 (364,464)* *CHO 7 (674,071)* *CHO 8 (592,388)* *CHO 9 (581,486)*	Per 100,000 population* 1.8 0.7 1.3 1.7 1.2 0.3 0.1 1.2 1.7		

^$^Calculated using the Community Healthcare Organisations Report [23]

### Clinical activities

As outlined in Table 2, most hospital-based podiatrists reported treating high-risk patients (H: n = 15; 65%, HC: n = 6;75%), providing a rapid access service (H: n = 17;74%, HC: n = 6;75%), treating patients with active foot disease (H: n = 18;78%, HC: n = 6;75%), educating patients on the risk of diabetes to the lower limb (H :n = 18;78%, HC: n = 6;75%) and recording activity statistics (H: n = 17;74%, HC: n = 6;75%). For those working across both the hospital and community settings (n = 8), data relating to their clinical activities in the community setting was missing for all (Table 2). Most community-based podiatrists reported treating non-diabetic foot pathologies (n = 14;74%), carrying out annual review of moderate risk patients (n = 14;74%) and high-risk patients (n = 12;63%) and referring patients with active foot disease to hospital podiatrists (n = 16;84.2%). No-one reported carrrying out an annual review of those at low risk. Few reported providing education on the risk of diabetes to the lower limb (n = 3;15.8%) and referring high risk patients to a hospital podiatrist (n = 6;31.6%). For those who provided a reason for not carrying out specific activities, responses are available in Table S2 in supplementary files.

### Providing structured education

Few reported providing structured education to allied healthcare professionals, including to GPs (H: n = 6;26%, C: n = 2;11%), public health nurses (H: n = 6;22%, C: n = 4;21%), nursing (H: n = 9;39%, C: n = 2;11%) and medical staff (H: n = 7;30%, C: n = 0) in hospitals, and allied healthcare professionals (H: n = 5; 22%; C: n = 0). Where respondents did provide structured education, it was typically provided through group education sessions (H: n = 13;57%, C: n = 7;37%) or a one-to-one session with the healthcare professional (H: n = 4;17%, C: n = 2;10.5%). As outlined in Table 2, data were missing for all participants (n = 8) who worked across both hospital and community settings.

**Table 2:** Clinical activities

	Hospital (n = 23) N, (%)	Community (n = 19) N, (%)	Hospital & Community (n = 8) N, (%)
Treat non-diabetic foot pathologies	5 (21.7)^¥^	14 (74)^¥^	6 (75)^§^
Refer patients with non-diabetic foot pathologies to community podiatry	12 (52.2)^¥^	Not asked	2 (25)^§^
Annual review of low-risk patients	Not asked	0%	Missing for all
Annual review of moderate risk patients	6 (26)^¥^	14 (74)^¶^	5 (62.5)^§^
Refer high risk pts to hospital podiatrist	Not asked	6 (31.6)^¶^	Missing for all
Annual review of high-risk patients	13 (56.5)^º^	Not asked	5 (62.5)^§^
Review high risk patients	Not asked	12 (63%)	Missing for all
Treat high risk patients	15 (65.2)^¥^	Not asked	6 (75)
Refer patients with active foot disease to hospital podiatrists	Not asked	16 (84.2)^¶^	Missing for all
Provide rapid access service	17 (74)^¥^	Not asked	3(37.5)^§^
Treat patients with active foot disease	18 (78.3)^¥^	Not asked	6 (75)^§^
Weekly review of patients with active foot disease until healed	17 (74)^¥^	Not asked	6 (75)^§^
Educate people with diabetes	18 (78.3)^¥^	3 (15.8)	6 (75)^§^
Record activity statistics	17 (74)^¥^	16 (84.2)^¶^	6 (75)^§^
**Providing Structured Education to Allied Healthcare professionals** ^ß^			
General Practitioners	6 (26)	2 (11)	0
Public Health Nurses	6 (22)	4 (21)	0
Nursing staff in hospitals	9 (39)	2 (11)	0
Medical staff in hospitals	7 (30)	0	0
Allied healthcare professionals	5 (22)	0	0
Do not provide structured education	3 (13)	3 (16)	0
Missing	6 (30)	4 (26)	0

^¥^Missing data for five respondents; ^§^Missing data for two respondents; ^¶^Missing for three respondents; ºMissing for six respondents; ^ß^As participants ticked a box if they provided education to these groups, and so we could not calculate missingness

### Use of screening tools

As outlined in Table S3 in supplementary files, twenty-five (50%) respondents reported using the recommended diabetic foot screening tool, including 52% (n = 12) of those based in the hospital only, 47% (n = 9) of those based in the community only and 50% (n = 4) of those working within both settings. Those who did not use this tool, reported either using a locally developed tool (H: n = 2;9%, C: n = 2;10.5%, HC: n = 2;25%), not using a specific screening tool (H: n = 1;4%; C: n = 0, HC: n = 0), or not carrying out screening (H: n = 3;13%, C: n = 0, HC: n = 0).

### Multidisciplinary team members and referral access

**Fig. 1:**
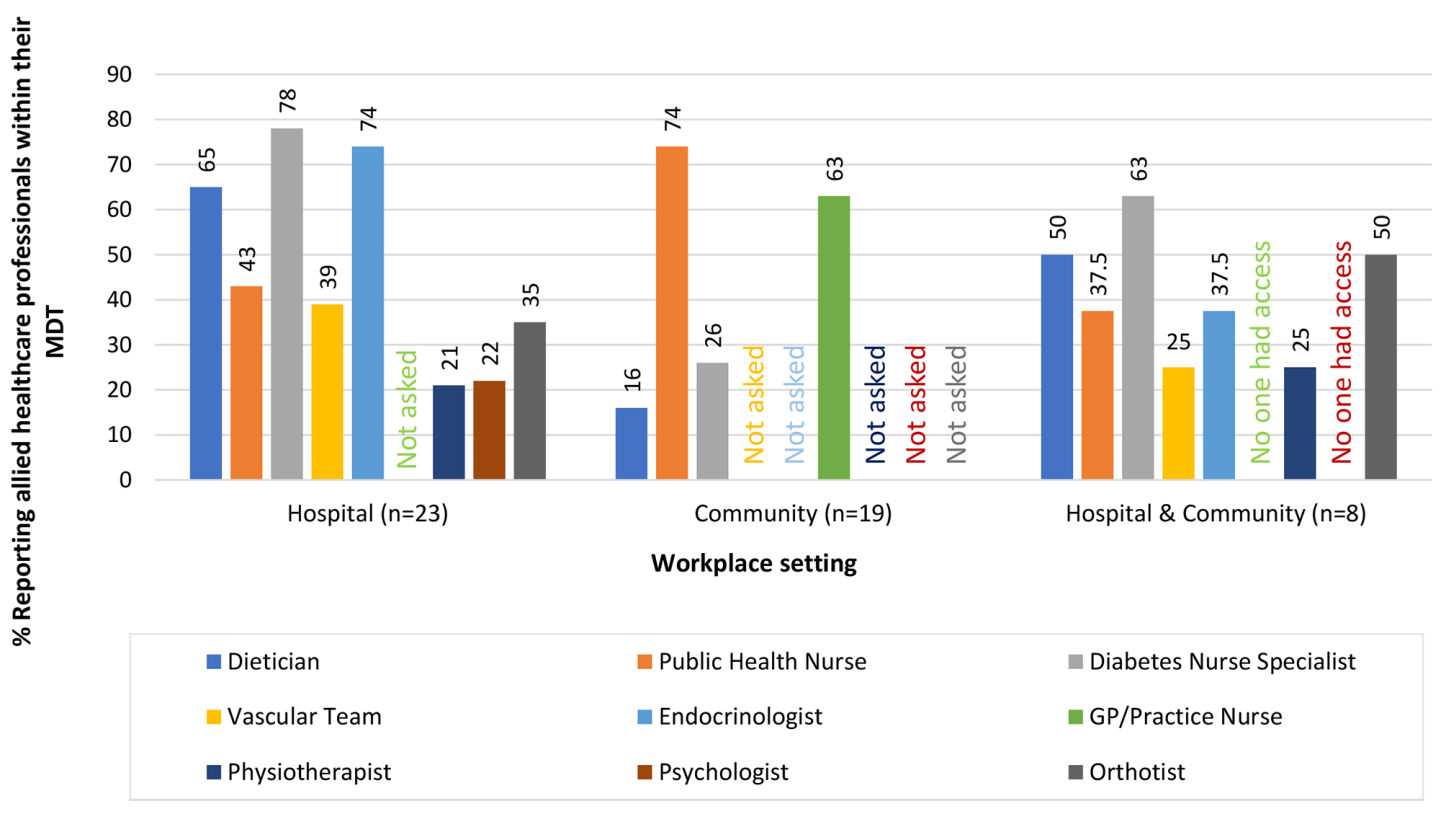
Members of the multidisciplinary team within podiatrists’ workplace setting

**Fig. 2:**
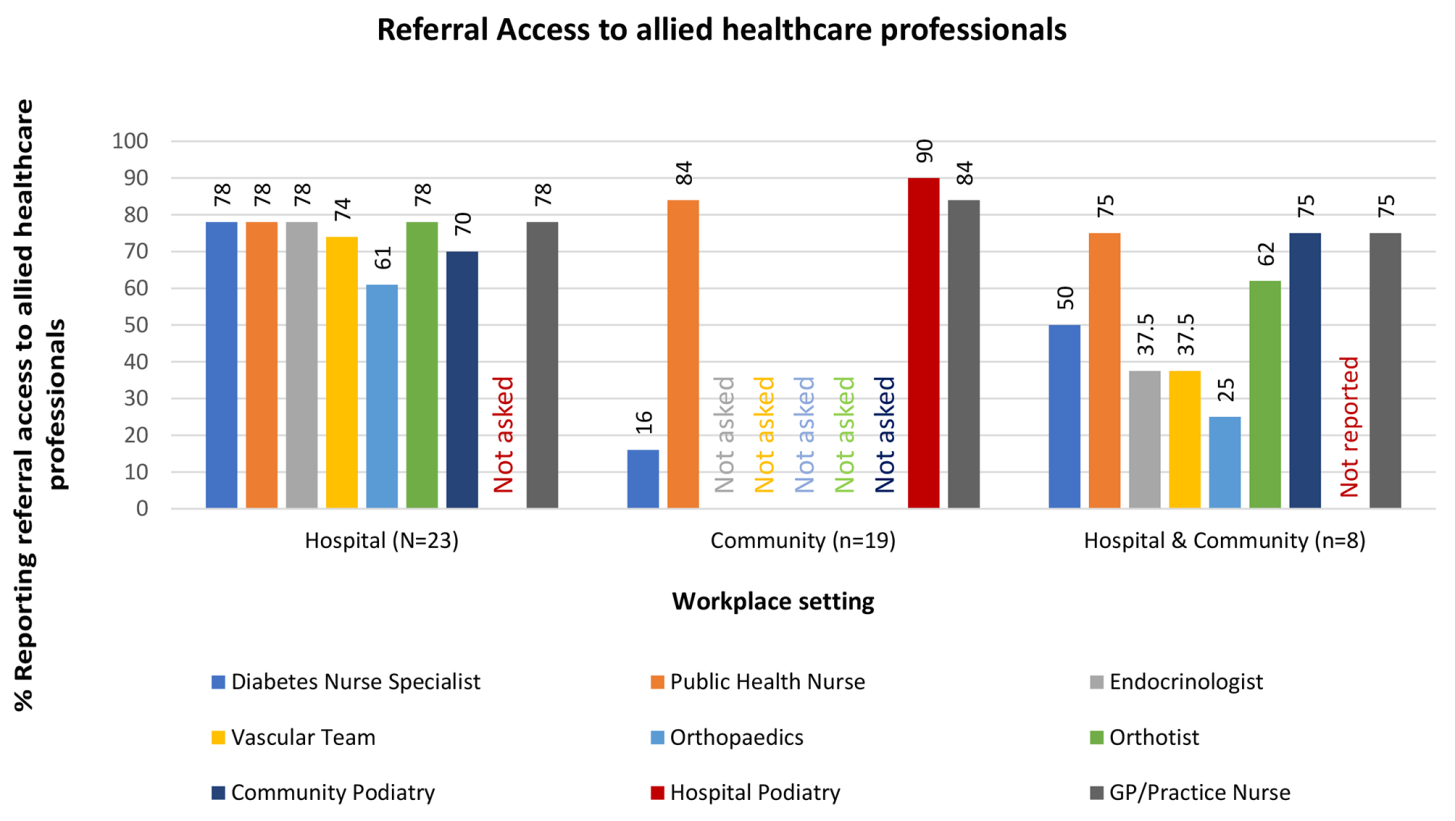
Referral access to allied healthcare professionals

Participants reported who the multidisciplinary team (MDT) members in their workplace were (Fig. 1), and who they had referral access to (Fig. 2). Most hospital-based respondents had access to a dietician (H: n = 15;65%, HC: n = 4;50%), diabetes nurse specialist (H: n = 18;78%, HC: n = 5;63%), and endocrinologist (H: n = 17;74%, HC: n = 3;37.5%), as part of their MDT. Few had a vascular team (H: n = 9;39%, HC: n = 2;25%), physiotherapist (H: n = 5;21%, HC: n = 2;25%), psychologist (H: n = 5;22%, HC: n = 0), and orthotist (H: n = 8;35%, HC: n = 4;50%) within their MDT. However, as outlined in Fig. 2, many hospital-based podiatrists had referral access to these services. As outlined in Fig. 1, podiatrists working in the community setting only had greater access than those working across community and hospital settings to a public health nurse (C: n = 14;74%, HC: n = 3;37.5%) and a GP/practice nurse (C: n = 12; 63%;HC: n = 0). However, they had inferior access to a diabetes nurse specialist within their own team (C: n = 5;26%, HC: n = 5; 63%) and referral to other teams (C: n = 3;16%, HC: n = 4;50%).

### Satisfaction with hospital and community services

Hospital-based podiatrists were satisfied with referral pathways used by GPs and practice nurses (H: n = 10; 43%, HC: n = 2; 25%) and community podiatrists (H: n = 15; 65%, HC: n = 3;37.5%), and screening by community podiatrists (H: n = 12; 52%, HC: n = 3;37.5%). Most community-based podiatrists reported being dissatisfied with screening (C: n = 9; 47%, HC: n = 0) and management of low-risk patients (C: n = 9;47%, HC: n = 2;25%) in the GP setting. See Table S4 in supplementary files for further information.

### Continuous professional development

Data on CPD was missing for all (n = 8) participants working across both setting. Few reported having a protected budget (H: n = 5; 21%, C: n = 0) and protected time (H: n = 6;26%, C: n = 3;16%). Activities most frequently engaged included conference attendance (H: n = 11; 48%, C: n = 12; 63% ), educational courses (H: n = 12; 52%, C: n = 5;26%), reading journal articles (H: n = 12;52%, C: n = 11;58% ) and undertaking learning on HSELand (www.HSELand.ie) (H: n = 8; 34%, C: n = 9;47%). More information is available in Table S5, in supplementary files.

### Qualitative: experiences of implementing the Model of Care

Responses (n = 33; 66%) to the open-ended question highlighted positive and negative experiences of implementing the Model of Care. Of those who responded, eighteen (56%) highlighted negative experiences, and twenty-five (78%) highlighted changes that need to be made. Five (16%) highlighted positive experiences, but all also reported either negative experiences or changes that need to be made.

Negative experiences were categorised as (1) lack of podiatry manager, (2) lack of resources (3) lack of awareness amongst allied healthcare professionals, (4) lack of integration between hospital and community podiatry services and (5) lack of career progression. Those working across community and hospital settings felt that the **lack of a dedicated podiatry manager** presented a challenge for successful implementation, as podiatry was *“not represented at senior management meetings”* (ID#3), and there was a lack of leadership and governance for podiatry services. In addition, one respondent noted how, because they had to attend meetings or write clinical policies, they were unable to maintain clinical responsibilities.

A **lack of resources** was also reported as a challenge, including a shortage of podiatrists (n = 8), lack of administrative support (n = 4) and building infrastructure (n = 3), as they could not find “*appropriate clinic rooms to provide treatment” (ID#12)* and *“due to on-going lack of resources and any definitive infrastructure it is bordering on being unsafe”* (ID#7). While these challenges were flagged across different regions and settings, there was a sense that it was a particular issue in places where the service was completely new:

*“Very poor so far. Started community post where no previous service was ever before. Difficulty in finding appropriate clinic rooms. Push back from nurses in health centres. Push back from Podiatrists in hospital wanting me to do all their work and refusal to accept active pts if I don’t accept their patients”* (ID#22).

*“Upon first taking up my current post the ‘DM Podiatrist’ based in the [redacted] hospital was a [redacted] with no proper DM referral pathways in place - I had to develop policies/procedures/referral criteria”* (ID#16).

Some respondents (n = 8) felt the Model of Care could not be implemented effectively because insufficient numbers of podiatrists were being employed to meet service demands. In one region, only one community podiatrist was employed, seeing only ‘*active non-diabetic wounds’* (ID #3), meaning those at moderate risk and high risk for ulceration were not being treated and managed in line with recommendations. Elsewhere, one respondent noted they have been employed in the HSE for several years and felt the Model of Care was becoming increasingly difficult to implement due to insufficient numbers of podiatrists to meet service demands, citing a lack of awareness amongst allied healthcare professionals on the role of podiatry and inappropriate use of diabetic foot care pathways as adding to this challenge. This was reported across each CHO, with at least one respondent from each CHO reporting a lack of awareness amongst allied healthcare professionals about the role of podiatrists (n = 6), inappropriate use of referral pathways (n = 5) and of inappropriate management of diabetic foot emergencies (n = 4). In addition,  one respondent flagged how in one case this led to a potentially preventable limb loss. To raise awareness, some (n = 7) flagged the need for *“Mandatory Teaching for all consultant teams, department and GP practices”* (ID #8).

Another challenge noted (n = 10) was the **lack of integration between hospital and community podiatry services.** Few provided a reason for this, but two noted that from the outset, local processes and pathways were not in place to support development and integration where new podiatry services were established. Some (n = 4) felt there was a **lack of career progression** available to podiatrists, as specialist’s role are not often available and there is often no “*progression available within the team*” meaning “*mapping and development of the service does not reach its full potential and the podiatrists are in general demoralised*” (ID#1). In addition, some noted how there is a lack of recognition of post-graduate degrees and ability to use skills to enhance implementation of the Model of Care, with some having colleagues that had either left, or were looking to leave, the podiatry profession.

Finally, few (n = 7, 22%) reported positive experiences of providing diabetic foot care since the introduction of the Model of Care. These were more general comments, with only two community podiatrists within the same CHO outlining specific positive experiences: increased awareness of the risk of diabetes to the lower limb and appropriate use of referral pathways.

Twenty-five (78%) identified necessary changes to address reported challenges. These included the need for a podiatry manager per CHO (n = 3), more provision of podiatry posts (n = 4), greater integration between hospital and community services (n = 7), increased training on the role of podiatry in diabetes care and appropriate use of referral pathways amongst allied healthcare professionals (n = 11) including those within the community setting (n = 3) and A&E setting (n = 2) and support for career progression and mentorship (n = 4). Some noted the need for changes that had not been flagged as challenges, including changes to the screening tool so it is less unwieldy (n = 6) and improvement to footwear and orthotic services (n = 4) to reduce waiting lists and high costs to the health service.

## Discussion

Previous evidence has shown that there is often a gap between what is set out by a policy and what it looks like when delivered to service users. In addition, other have noted the importance of gaining the perspectives of those involved in implementing policy to ensure its success. This study set out to explore whether podiatrists’ work activities align with national recommendations, and their experiences of providing diabetic foot care since introduction of a model of care, with the aim of identifying barriers to service delivery and areas for improvement. Although, little previous research exists on podiatrists’ experiences of providing integrated diabetic foot care [13], affording limited comparisons with the international situation, we identified specific areas for attention that stakeholders involved in diabetic foot care policies need to consider to enable successful implementation of evidence and policies into practice [7, 13].

First, we found that the majority of the respondent’s patients had a diagnosis of diabetes, and most were performing clinical activities in line with Model of Care recommendations. For those who responded to the open-ended question on reasons for not doing specific clinical activities, the most common reason cited was that they did not have enough podiatrists to meet service demands. Insufficient numbers to meet service demands is in keeping with findings internationally [13, 22]. As outlined in their recently published systematic review, McPherson and colleagues identified inadequate staff numbers as a barrier for patients in accessing footcare, with rural areas being worse affected [22]. However, little is known about the specific reasons for inadequate staffing levels internationally [13]. The identified need for training and professional development to support career progression and maintain staff morale could be one reason, as some respondents reported that some podiatrists were looking to leave the profession due to lack of career progression and lack of resources. We did find that there were significantly more senior podiatrists in comparison to clinical podiatry specialists and podiatry clinical managers, with there being no clinical specialist in the community setting and no podiatry manager in the hospital setting, and some noting there were no opportunities for progression to these roles. Elsewhere, evidence suggests that the number of people enrolling in podiatry courses is declining in some regions, with staff retention being an ongoing issue internationally amongst podiatrists and healthcare staff in general [22, 26–28]. Integrated diabetic foot care programmes cannot be successful without enough podiatrists, and so strategies are needed to support career progression and maintain staff retention. Others have noted that reasons for poor healthcare staff retention is multifactorial, with some reasons including poor management and insufficient access to professional development and career opportunities, and we found the same in our current study [29]. This is of concern to those involved in implementing integrated diabetic foot care programmes if they want to ensure there is a skilled podiatry workforce to meet service demands to prevent ulceration and amputation.

Results also show a lack of integration between hospital and community podiatry services, although we do not know exactly what aspect of integration failed. One potential reason could be the fact that some community health organisations have podiatrists in the hospital setting but none in the community setting, or vice versa, meaning there could be a very good hospital or community service but no one in the corresponding setting to support integrated care. Another reason could be because of the reported lack of awareness of the role of podiatry and use of appropriate referral pathways amongst allied healthcare professionals, which was reported to lead to misdiagnosis and inappropriate referrals. However, it is important to note that few reported providing structured education to allied healthcare professionals within community and hospital-based settings. One reason for this could have been because of insufficient numbers of podiatrists to carry out clinical duties, and so they could not afford the time to provide education. In describing the implementation of a combination of strategies aimed at spreading integrated diabetic footcare in Tuscany, Nuti et al. found clinicians (including podiatrists, endocrinologists, nurses) reported that a lack of information flow and coordinated care between healthcare professionals within the same institutions and between different institutions was a barrier to integrated diabetic foot care [7]. This lack of integration may also contribute to the fact that not every podiatrist had access to members of the MDT, either within their own setting or to a different setting, despite people with diabetes accounting for the majority of their client load. A fundament of integrated care is that different services and organisations work together efficiently to promote ease of access to, and navigation of, health services for patients.

The lack of integration identified within the current study may have been compounded by the fact that when the Model of Care was introduced, podiatry services were put in place where there had never been services before, as two respondents did note that where new services were established, processes and pathways were not put in place to support development and integration between hospital and community services, potentially leading to conflict. While this change was welcome, in line with previous policy recommendations in Ireland, results from the current study and others suggest that the necessary organisational infrastructure was not in place to support integration between hospital and community services [13, 30]. Other potential reasons for the lack of integration, may have been due (1) the reported lack of podiatry managers and (2) the lack of awareness of the role of podiatry and use of appropriate referral pathways amongst allied healthcare professionals, leading to misdiagnosis and inappropriate referrals, that was highlighted by respondents.

Finally, we note that, since this study was conducted, an updated Model of Care was published to reflect changes in Ireland’s wider health system [14, 31, 32]. Alongside its publication, some of the key challenges we identified were addressed, including (1) development of a training module on diabetic foot screening and referral pathways available to all healthcare professionals working within Ireland on the online training tool HSELand (www.hseland.ie), (2) revision of the diabetic foot screening tool so it allows for appropriate risk categorisation, (3) employment of podiatry managers for each CHO and (4) a recruitment drive for podiatrists to advance implementation of recommendations outlined within the Model of Care for the Diabetic Foot [31]. However, little is known yet about the success of these initiatives. Nonetheless, our study still highlights areas of attention for those involved in decisions on integrated diabetic foot care programmes internationally. Also, before a new service is implemented, it highlights preparatory work is needed to better support clinicians and integrated care. First, they need to make there are enough podiatrists in place in both the hospital and community setting to meet service needs. Second, with increased staffing comes a need for more clinical resources including administration and infrastructure, and if these are not in place before podiatrists take up their posts, they may be unable to fulfil their clinical role. Third, allied healthcare professionals within, and outside organisations need to be made aware of the role of podiatrists and appropriate referral pathways ensure those who need specialist input are seen at the right time and in the right place. Finally, relevant stakeholders need to support, and recognise continuous professional development, to support career progression, maintain staff morale, and ultimately there is a skilled workforce in place.

### Strengths and limitations

This study is the first to focus explicitly on hospital and community-based podiatrists’ experiences of an integrated diabetic foot care programme in Ireland. The first strength is that it corroborates the findings of a previously published realist evaluation which using semi-structured interviews provided insight into a small sample (n = 6) of hospital-based podiatrists and their experiences of providing integrated diabetic foot care [13]. The second strength is the use of questionnaires previously used in the UK, and consulting with the national lead for podiatry at the time, allowing us to tailor questions and responses to the Irish context. Finally, our open-ended question on podiatrists’ experiences of implementing the Model of Care and the changes they would make provides insight into whether experiences align with model recommendations.

However, a limitation of the survey design is that we could not explore certain concepts in more detail. For example, few community-based podiatrists reported educating people with diabetes on the risk of diabetes on the lower limb, but we do not know why this is. In addition, although we obtained information on podiatrist’s satisfaction with services, we do not know why they are unsatisfied with certain elements of care. Future interviews or focus group discussions could be beneficial to explore these issues in greater depth. A high level of missing data from those who worked across the hospital and community setting, may have arisen from the survey design as they were provided response options relating to both settings which may have been burdensome and resulted in non-completion of some questions. We also recognise that this study only provides an insight into one profession involved in the Model of Care for the Diabetic Foot, albeit one that is key to delivery of successful diabetic foot care. Finally, it is important to acknowledge that these results are being published five years after data collection, and two years after the introduction of an updated version of the Model of Care. However, recommendations for diabetic foot screening and management have not differed significantly between the 2011 and 2021 versions of the Model of Care for the Diabetic Foot. Screening for risk factors remain within the GP setting, however, a new funding model has been introduced which entitles all people with a diagnosis of diabetes and who hold a medical card (access to this depends on annual income) to one free diabetic foot screening per year. Management of the at-risk foot remains within the care of community-based podiatrists, however, these podiatrists should now be situated within foot protection teams consisting of GPs, practice nurses, diabetes nurse specialist, orthotists, and administrative staff. Those with active foot disease will still be seen by multidisciplinary diabetic foot teams (MDFT), which are based primarily within the hospital setting. However, to better support integrated hospital and community diabetic foot care, some MDFT review clinics will take place in the community setting [31].

## Conclusion

The aim of the Model of Care for the diabetic foot was to establish a system of integrated care where primary, secondary, and tertiary services work together to communicate effectively and coherently to manage people with diabetes. Our results indicate this did not happen in every setting, as of 2018. While our study provides novel insights into experiences of podiatrists, it also highlights that it is simply not enough to just develop a policy but there needs to systematic examination of how policies are implemented with input from relevant stakeholders.

### Electronic supplementary material

Below is the link to the electronic supplementary material.

Supplementary Material 1

Supplementary Material 2

## Data Availability

The dataset is available from the corresponding author (email: jpallin@ucc.ie) upon reasonable request.

## Abbreviations

NCPD: National Clinical Programme for Diabetes
