# Supplementary material for: Implementing an integrated diabetic foot care programme in Ireland: podiatrists’ experience
Source: BMC Health Serv Res. 2023 Oct 26;23:1157. doi: 10.1186/s12913-023-10144-z (PMC10601248; doi:10.1186/s12913-023-10144-z)
Supplement: Supplementary file 1 — Supplementary Material 1 [file 12913_2023_10144_MOESM1_ESM.docx]

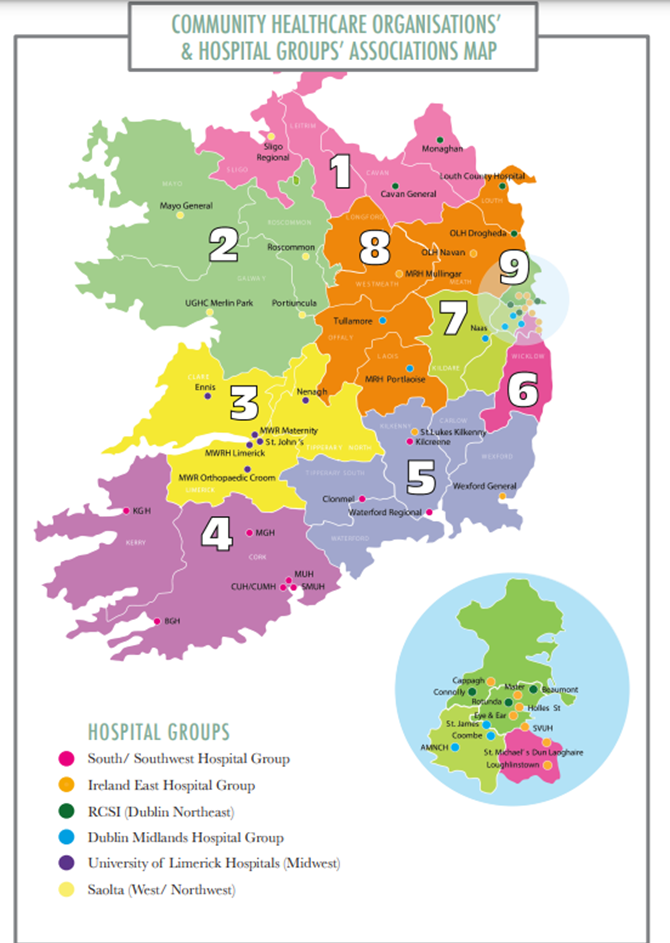


***Figure S1., Community healthcare organisations and hospital groups associations map. Available at:*** [***https://www.hse.ie/eng/services/publications/corporate/cho-chapter-1.pdf***](https://www.hse.ie/eng/services/publications/corporate/cho-chapter-1.pdf)

| ***Table S1. Community Health Organisation and area it covers*** | |
| --- | --- |
| Community Health Organisation (CHO) Number | Area |
| 1 | **Donegal, Sligo/Leitrim/West Cavan, Cavan/Monaghan** |
| 2 | Galway, Roscommon, Mayo |
| 3 | Clare, Limerick, North Tipperary / East Limerick |
| 4 | Kerry, North Cork, North Lee, South Lee, West Cork |
| 5 | South Tipperary, Carlow / Kilkenny, Waterford, Wexford |
| 6 | Wicklow, Dun Laoghaire, Dublin South East |
| 7 | Kildare / West Wicklow, Dublin West, Dublin South City, Dublin South West |
| 8 | Laois / Offaly, Longford / Westmeath, Louth, Meath |
| 9 | Dublin North Central, Dublin North West |

| **Table S2, Reasons for doing certain clinical activities** |
| --- |
| **Treat non-diabetic foot pathologies** |
| **Hospital only based podiatrists.**   - *“Only patients with diabetes active ulcerations are referred to the hospital service here”* (ID# 1) - *“Diabetic only service. Terms of contract”* (ID# 3) - *“not part of my job description” (ID# 32)* - *I work in a level 4 acute hospital (ID# 5)* - *Don't see non diabetic patients. Do treat diabetic patients with these problems (ID# 6)* - *in model 4 hospital, active and high risk diabetic patients only (ID# 7)* - *Often managed by the community Podiatry Team (ID# 8)* - *we only treat diabetic foot pathologies (ID# 9)* - *the service is for diabetes pateints with high active foot disease (ID# 10)* - *not appropriate (ID# 11)*   **Community only based podiatrists**   - *do not treat non-diabetic - non active feet (ID# 38)* |
| **Refer patients with non-diabetic foot pathologies to community podiatry** |
| **Hospital only based podiatrists:**   - There is no public community podiatrist in my area (ID# 5) - Don't see non diabetic patients. Do treat diabetic patients with these problems (ID# 6) - we only treat diabetic foot pathologies - non pathologies would be referred to community podiatry by the team members in OPD via their GP's (ID# 9) |
| **Annual review of low-risk patients** |
| **Community only based podiatrists.**   - understaffed - not seen in department (ID# 13) - Completed by practise nurses (ID# 14) - Low risk screening is to be done by Nursing Staff (ID# 16) - Low risk referrals not accepted (ID# 17) - do not see Low Risk patients (ID# 38) - do not accept low risk patient referrals (ID# 21) - we do not see low risk patients (ID# 48) - Do Not See as Screened by GP/PN/PHN (ID# 49) |
| **Annual review of moderate risk patients** |
| **Hospital only based podiatrists.**   - Too many active wounds for this. (ID# 3) - only deal with active and high risk (ID# 4) - I work in a level 4 acute hospital (ID# 5) - Do on assessment but then transfer to community Podiatry (ID# 6) - moderate risk seen in community not model 4 hospital (ID# 7) - we don't perform annual review - these happen in the diabetes OPD with onward referral to us for acute conditions (ID# 9)   **Community only based podiatrists.**   - Cannot meet the annual review target based on caseload and staff levels (ID# 17) - we do not see moderate risk patients (ID# 48) - Insufficient staff at present due to non filled posts and suspension of service for Moderates (ID# 49) |
| **Annual review of high-risk patients** |
| **Hospital only based podiatrists.**   - Where possible (ID# 3) - we don't perform annual review - these happen in the diabetes OPD with onward referral to us for acute conditions (ID# 9) - Often managed by the community Podiatry Team (ID# 8) - should be carried out in community podiatry (ID# 11) |
| **Refer high risk pts to hospital podiatrist** |
| **Community only based podiatrists.**   - Only refer active foot disease patients to Hospital Podiatrist as per HSE Model of Care of Diabetic Foot (ID# 15) - Generally do not refer high risk pts unless they have ulcer (ID# 16) - The hospital podiatrist will not accept high risk referrals. (ID# 17) - If not ulcerated do not refer these patients to DFPS (ID# 18) - only those with active foot disease (ID# 20) - High risk patients kept in community in my area (ID# 38) - Patients easily managed in community (ID# 22) - the hospital podiatrist only accepts patients with active foot ulcerations (ID#48) |
| **Weekly review of AFD until healed.** |
| **Hospital only based podiatrist.**   - Review of active foot disease may not be weekly - review based on clinical need (ID# 11) |
| **Record activity statistics** |
| **Hospital only based podiatrist.**   - Not set up yet but starting (ID# 3) - Currently only recording numbers of patient contacts under each risk level. Cannot afford to give needed time to gathering/manipulating statistics as it would negatively impact the running of our clinics and the number of clients we would be able to see. (ID# 28) |

| **Table S3, Responses to questions on screening tools used** | | | |
| --- | --- | --- | --- |
|  | Hospital (n=23)  N (%) | Community (n=19)  N (%) | Hospital and Community (n=8)  N (%) |
| National Diabetic Foot Screening Tool | 12 (52) | 9 (47) | 4 (50) |
| Locally Developed Tools | 2 (9) | 2 (11) | 2 (25) |
| Does not use a specific tool | 1 (4) | 0 | 0 |
| Do not provide diabetic foot screening | 3 (13) | 0 | 0 |
| Missing | 5 (22) | 8 (42) | 2 (25) |

| Table S4, Satisfaction with community and hospital based services | | | | | |  |
| --- | --- | --- | --- | --- | --- | --- |
| Parameter | **Respondent work setting** | **Satisfied** | **Neither satisfied nor unsatisfied** | **Dissatisfied** | **I don’t know if this happens** | **No response** |
| Appropriate screening by GP^a^/PN^b^ | *Hospital (N=23)*  *Community(N=19)*  *Hospital/Community (N=8)* | 13% (n=3)  5% (n=1)  25% (n=2) | 26% (n=6)  21% (n=4)  12.5% (n=1) | 17% (n=4)  48% (n=9)  0 | 26% (n=6)  10% (n=2)  25% (n=2) | 22% (n=5)  16% (n=5)  37.50% (n=3) |
| Low risk patients managed by GP | *Hospital*  *Community*  *Hospital/Community* | 17% (n=4)  11% (n=2)  25% (n=2) | 13% (n=3)  21% (n=4)  0 | 22% (n=5)  47% (n=9)  0 | 30% (n=7)  5% (n=1)  25% (n=2) | 22% (n=5)  16% (n=3)  50% (n=4) |
| PN uses the national screening tool | *Hospital*  *Community*  *Hospital*  */Community* | 35% (n=8)  16% (n=3)  12.5% (n=1) | 9% (n=2)  11% (n=2)  0 | 26% (n=6)  26% (n=5)  25% (n=2) | 13% (n=3)  5% (n=1)  0 | 22% (n=5)  42% (n=8)  62.5% (n=5) |
| PHNs^c^ use the national screening tool | *Hospital*  *Community*  *Hospital*  */Community* | 17% (n=4)  5% (n=1) | 0  21% (n=4) | 0  43% (n=10) | 0  11% (n=2) | 87% (n=20)  11% (n=2)  100% (n=8) |
| Appropriate referral pathways by GP/PNs | *Hospital*  *Community*  *Hospital*  */Community* | 44% (n=10)  Not asked  25% (n=2) | 17% (n=4)  Not asked  0 | 22% (n=5)  Not asked  12.5% (n=1) | 0  Not asked | 22% (n=5)  Not asked  62.5% (n=5) |
| Appropriate screening by community podiatrists | *Hospital*  *Community*  *Hospital*  */Community* | 52% (n=12)  Not asked  37% (n=3) | 4% (n=1)  Not asked  0 | 17% (n=4)  Not asked  12% (n=1) | 9% (n=2)  Not asked  0 | 22% (n=5)  Not asked  50% (n=4) |
| Appropriate referral pathways by community podiatrists | *Hospital*  *Community*  *Hospital*  */Community* | 65% (n=15)  Not asked  37% (n=3) | 4% (n=1)  Not asked  0 | 13% (n=3)  Not asked  12% (n=1) | 0  Not asked  0 | 22% (n=5)  Not asked  50% (n=4) |
| Annual review of moderate risk pts completed by community podiatrists | *Hospital*  *Community*  *Hospital*  */Community* | 35% (n=8)  Not asked  38% (n=3) | 0  Not asked  0 | 35% (n=8)  Not asked  12.5% (n=1) | 22% (n=5)  Not asked  12.5% (N=1) | 26% (n=6)  Not asked  38% (n=3) |
| High risk foot patients are reviewed by hospital podiatrist | *Hospital*  *Community*  *Hospital*  */Community* | Not asked  16% (n=3)  0 | Not asked  32% (n=6)  0 | Not asked  32% (n=6)  0 | Not asked  5% (n=1)  0 | Not asked  16% (n=3)  100% (n=8) |
| Active foot disease patients are seen by a hospital podiatrist within 24hours | *Hospital*  *Community*  *Hospital*  */Community* | Not asked  52% (N=8)  0 | Not asked  11% (n=2)  0 | Not asked  22% (n=6)  0 | Not asked  0  0 | Not asked  16% (n=3)  100% (n=8) |
|  |  |  |  |  |  |  |
| ^a^GP – General Practitioner; ^b^PN – Practice nurse; ^c^PHNs – Public Health Nurses | | | | | | |

| Table S5., Continuous professional activities | | | |
| --- | --- | --- | --- |
|  |  |  |  |
|  | Hospital (n=23)  N (%) | Community (n=19)  N (%) | Hospital & Community (n=8)  N (%) |
| Protected Budget for CPD | 5 (22) | 0 | 0 |
| Protected time for CPD | 6 (16) | 3 (16) | 0 |
| **Activities engaged in:** | | | |
| Conference attendance | 11 (48) | 12 (63) | 0 |
| Attend national CPD days organised by NCPD | 7 (30) | 3 (16) | 0 |
| Attend national CPD days (not facilitated by NCPD) | 2 (9) | 3 (16) | 0 |
| Educate other professionals about podiatry | 8 (35) | 7 (37) | 0 |
| Educational/upskilling courses | 12 (52) | 5 (26) | 0 |
| Educational/upskilling workshop | 9 (39) | 6 (32) | 0 |
| None | 0 | 0 | 0 |
| **Self-directed CPD activities** | | | |
| Review journal articles/literature | 12 (52) | 11 (58) | 0 |
| Watch webinars | 4 (14) | 3 (16) | 0 |
| Undertake learning on HSELand | 8 (35) | 9 (47) | 0 |
| Reflective practice | 7 (30) | 6 (32) | 0 |
| I do not engage in these activities | 1 (4) | 0 | 0 |
