# Supplementary material for: Implementing an integrated diabetic foot care programme in Ireland: podiatrists’ experience
Source: BMC Health Serv Res. 2023 Oct 26;23:1157. doi: 10.1186/s12913-023-10144-z (PMC10601248; doi:10.1186/s12913-023-10144-z)
Supplement: Supplementary file 2 — Supplementary Material 2 [file 12913_2023_10144_MOESM2_ESM.pdf]

Consent

We want to gain a greater understanding of your role in diabetes care. This study will improve the understanding of how podiatry services are currently organised in Ireland. This information is important to inform plans for changes in diabetes management in Ireland.

* 1. Before proceeding with the survey, we ask you to read the following and indicate your consent below.

- The purpose and nature of the study has been explained to me in writing.
- I am participating voluntarily.
- I understand that I can withdraw from the study, without repercussions, at any time.
- I understand that I can withdraw permission to use the data, in which case the material will be deleted.
- I understand that anonymity will be ensured in the report and any subsequent publications.

○ I consent to patriciate in the National Survey of Podiatrists in Ireland

Demographic Information

* 2. Please indicate the following information:

Name:

Workplace Name(s):

Workplace Location(s) (City/Town):

Community Healthcare Organisation (CHO) Number (if applicable):

* 3. What is your current job title? (Select all that are appropriate)

- [ ] Staff Grade Podiatrist
- [ ] Senior Podiatrist
- [ ] Assistant Clinical Specialist
- [ ] Clinical Specialist
- [ ] Other (please specify)
- [ ] Podiatry Manager
- [ ] Podiatrist (Private Practice)
- [ ] Chiropodist

* 4. Please indicate your age group

* 5. What is your highest level of education relating to podiatry:

- ○ Higher Diploma
- ○ Undergraduate Degree
- ○ Postgraduate Degree (Master Degree)
- ○ Other (please specify)
- ○ Postgraduate Degree (PhD)
- ○ No formal education in podiatry

6. If applicable, in what educational institution did you complete your professional training in podiatry?

* 7. Please indicate the following information:

Years working as a podiatrist / chiropodist

Years providing foot care to people with diabetes

Year of uptake of your current post

* 8. Please indicate, using the options provided below, the professional association of which you are a member

- Society of Chiropodists and Podiatrists of Ireland
- Institute of Chiropodists and Podiatrists (Irish branch)
- Irish Chiropodists / Podiatrists Organization Ltd
- I am not a member of a professional association

* 9. This survey distinguishes between community settings, hospital settings and a private practice settings. Taking this into account, in what location(s) do you work?

- I work in a hospital only
- I work in the community only (excluding private practice)
- I work in private practice only
- I work in a hospital and in the community (excluding private practice)
- I work in a hospital and in private practice
- I work in the community and in private practice

Employment Details (Hospital)

* 10. Who are you employed and funded by?

	Employed by	Funded by
HSE	☐	☐
Community Hospital	☐	☐
Voluntary hospital	☐	☐
Other	☐	☐

Other (please specify)

* 11. Please indicate the hospital model:

○ Model 2 ○ Model 3 ○ Model 4 ○ Don't know

* 12. What is the whole time equivalent (WTE) of your position? (e.g. 0.5, 0.75, 1)

* 13. What percentage (%) of your client load are people with diabetes?

* 14. How did you answer the above question?

○ Actual data ○ Estimation ○ Actual data and estimation

Providing Diabetes Foot Care (Hospital)

* 15. Who are the multidisciplinary team members for diabetic foot care in your workplace?

- ☐ Dietitian
- ☐ Occupational Therapist
- ☐ Orthotist
- ☐ Physiotherapist
- ☐ Public Health Nurse
- ☐ Endocrinologist
- ☐ Psychologist
- ☐ Vascular Team
- ☐ Diabetes Nurse Specialist
- ☐ Other (please specify)

* 16. Please indicate the percentage (%) of clients referred to you from the following sources (from January 2017):

Self-referral

GP/Practice Nurse

Public Health Nurse

Diabetes Nurse Specialist

Community Podiatrist

Emergency Department

Internal (hospital) referral

* 17. How did you answer the above question?

- ○ Actual data
- ○ Estimation
- ○ Actual data and estimation

* 18. Please indicate the percentage (%) of inappropriate referrals you have received in the last month:
(Inappropriate referrals are those which fall outside of your treatment responsibilities, according to the National Footcare Model and/or your job specification)

* 19. How did you answer the above question?

- ○ Actual data
- ○ Estimation
- ○ Actual data and estimation

20. If you receive inappropriate referrals, please indicate the main reasons for this:

* 21. On average, what percentage (%) of your clients are:

Moderate Risk

High Risk

Active Foot Disease

Other (please specify)

* 22. How did you answer the above question?

- Actual data
- Estimation
- Actual data and estimation

* 23. Do you have referral access to the following services?

	I have referral access where I work	I have referral access to another hospital	I do not have referral access
Diabetes Nurse Specialist			
Endocrinologist/Diabetologist			
Vascular Surgery			
Orthopaedic Surgery			
Orthotists			

* 24. Do you have referral access to the following services in the community?

	Yes	No
Chiropodist		
GP/Practice Nurse		
Community Podiatrist (Public)		
Community Podiatrist (Private)		
Public Health Nurse		

Other (please specify)

* 25. In general, during your typical working week, do you carry out the following activities?

	Yes	No
Treat non-diabetic foot pathology such as nail conditions, corns, callous or verrucae	☐	☐
Refer patients with non-diabetic foot pathology such as nail conditions, corns, verrucae to the community podiatrist/chiropodist	☐	☐
Annual review of moderate risk patients	☐	☐
Annual review of high risk patients	☐	☐
Treat high risk patients	☐	☐
Provide a rapid access service (i.e. see referrals for active foot disease within 24 hours)	☐	☐
Treat patients with active foot disease	☐	☐
Weekly review to patients with active foot disease until the foot is healed	☐	☐
Educate the patient in diabetes related foot care	☐	☐
Record activity statistics	☐	☐

Other (please specify)

[ ]

26. If you answered 'No' to any of the following statements, please indicate the reason(s) for this:

Treat non-diabetic foot pathology such as nail conditions, corn, callous, or verrucae	
Refer patients with non-diabetic foot pathology to the community chiropodist/podiatrist	
Annual review of moderate risk patients	
Annual review of high risk patients	
Treat high risk patients	
Provide a rapid access service (i.e. see referrals for active foot disease within 24 hours)	
Treat patients with active foot disease	
Weekly review to patients with active foot disease until the foot is healed	
Educate the patient in diabetes related foot care	
Record activity statistics	

* 27. Please indicate your satisfaction with community services in your locality:

	Very satisfied	Satisfied	Neither satisfied nor unsatisfied	Unsatisfied	Very unsatisfied	I don't know if this happens
Appropriate screening is conducted by GPs/Practice Nurse	☐	☐	☐	☐	☐	☐
Patients at low risk of diabetic foot disease are managed in a GP practice	☐	☐	☐	☐	☐	☐
Appropriate referral pathways are used by GPs/Practice Nurses	☐	☐	☐	☐	☐	☐
Appropriate screening is conducted by podiatrists working in the community	☐	☐	☐	☐	☐	☐
Appropriate referral pathways are used by podiatrists working in the community	☐	☐	☐	☐	☐	☐
Annual review of moderate risk patients is completed by the community podiatrist	☐	☐	☐	☐	☐	☐

* 28. What is the waiting period for patients accessing your service under the following categories:

Active Foot Disease

High Risk

Moderate Risk

* 29. How did you answer the above question?

- Actual data
- Estimation
- Actual data and estimation

Service Overview (Hospital)

* 30. What tool do you use to screen for diabetes foot disease?

- ○ National Diabetes Foot Screening Tool
- ○ I do not use a specific screening tool
- ○ Locally developed tool
- ○ I do not provide diabetic foot screening services
- ○ Other (please name)

* 31. On average, how many patients with diabetes do you see per week?

* 32. Of your clients who are people with diabetes, how many appointments each week are DNA or cancelled appointments?

* 33. How did you answer the above question?

- ○ Actual data
- ○ Estimation
- ○ Actual data and estimation

* 34. Do you provide out-of-hours diabetes consultations?

- ○ Yes
- ○ No

35. If yes, when are the out-of-hours sessions held in your area? (Tick all that apply)

- ☐ At weekends
- ☐ In the evenings
- ☐ Other (please specify)

* 36. Do you provide a drop-in service for patients?

- ○ Yes
- ○ No

* 37. Are you currently collecting data about the podiatry service?

- ○ Yes
- ○ No

38. If yes, what information is being collected?

* 39. Is this data shared?

- ○ Yes
- ○ No
- ○ I do not collect data about the podiatry service

40. If yes, who is collected data shared with?

* 41. Has your department developed PPGs (procedures, pathways, policies and guidelines)?

Yes No

42. If yes, have the PPPGs developed been validated?

Yes No

43. We value your insight into diabetes care. Please use the space provided to describe your experiences of implementing the National Model of Diabetic Foot Care. If applicable, what are the changes you would make to the National Clinical Programme for Diabetes?

Employment Details (Community)

* 44. Who are you employed and funded by?

	Employed by	Funded by
HSE	☐	☐
Diabetes Ireland	☐	☐
Local Diabetes Initiative	☐	☐
Community Hospital	☐	☐
Voluntary hospital	☐	☐
Other	☐	☐

Other (please specify)

* 45. What is the whole time equivalent (WTE) of your position? (e.g. 0.5, 0.75, 1)

* 46. In what location(s) do you work? (Tick all that apply)

☐ Primary Care Centre

☐ Sole GP Practice

☐ Other (please specify)

* 47. What percentage (%) of your client load are people with diabetes?

* 48. How did you answer the above question?

○ Actual data

○ Estimation

○ Actual data and estimation

Providing Diabetes Foot Care (Community)

* 49. Who are the established multidisciplinary team (MDT) members for diabetic foot care in your workplace?

- [ ] Dietitian
- [ ] Diabetes Nurse Specialist
- [ ] Public Health Nurse
- [ ] Practice Nurse
- [ ] GP
- [ ] Other (please specify)

* 50. In relation to diabetic foot disease, what percentage (%) of your clients fit the following categories:

Low risk

Moderate risk

High risk

Active Foot Disease

* 51. How did you answer the above question?

- ( ) Actual data
- ( ) Estimation
- ( ) Actual data and estimation

* 52. Please indicate the percentage (%) of clients referred to you from the following sources (since January 2017):

Self-referral

GP/Practice Nurse

Public Health Nurse

Diabetes Nurse Specialist

Hospital Podiatrist

Hospital Consultant

* 53. How did you answer the above question?

- ( ) Actual data
- ( ) Estimation
- ( ) Actual data and estimation

* 54. Please indicate the percentage (%) of inappropriate referrals you have received in the last month:*(Inappropriate referrals are those which fall outside of your treatment responsibilities, according to the National Footcare Model and/or your job specification)*

* 55. How did you answer the above question?

- ( ) Actual data
- ( ) Estimation
- ( ) Actual data and estimation

56. If you receive inappropriate referrals, please indicate the main reasons for this:

* 57. What is the waiting period for patients accessing your service under the following categories:

Active Foot Disease

High Risk

Moderate Risk

Low Risk

* 58. How did you answer the above question?

- Actual data
- Estimation
- Actual data and estimation

* 59. Do you have referral access to the following services:

	Yes	No
Chiropodist		
GP/Practice Nurse		
Hospital Podiatrist		
Diabetes Nurse Specialist		
Public Health Nurse		
Podiatrist (Private)		

Other (please specify)

* 60. In general, during your typical working week, do you carry out the following activities?

	Yes	No
Treat non-diabetic foot pathology such as nail conditions, corns, callous or verrucae	☐	☐
Diabetic foot care education	☐	☐
Screen and categorise risk of diabetic foot disease (i.e. low / mod / high)	☐	☐
Annual review of low risk patients	☐	☐
Annual review of moderate risk patients	☐	☐
Review high risk patients	☐	☐
Refer high risk patients to the hospital podiatrist	☐	☐
Refer patients with active foot disease to the hospital podiatrist	☐	☐
Record activity statistics	☐	☐

Other (please specify)

____

61. If you answered 'No' to any of the following statements, please indicate the reason(s) for this:

Treat non-diabetic foot pathology such as nail conditions, corn, callous, or verrucae __________

Diabetic foot care education __________

Screen and categorise risk of diabetic foot disease (i.e. low / mod / high) __________

Annual review of low risk patients __________

Annual review of moderate risk patients __________

Review high risk patients __________

Refer high risk patients to the hospital podiatrist __________

Refer patients with active foot disease to the hospital podiatrist __________

Record activity statistics __________

* 62. Please indicate your satisfaction with local podiatry services:

	Very satisfied	Satisfied	Neither satisfied nor unsatisfied	Unsatisfied	Very unsatisfied	I don't know if this happens
Appropriate screening is conducted by GPs/Practice Nurse	☐	☐	☐	☐	☐	☐
Practice Nurse uses the national screening tool	☐	☐	☐	☐	☐	☐
Patients at low risk of diabetic foot disease are managed in a GP Practice	☐	☐	☐	☐	☐	☐
Public health nurses use the national screening tool	☐	☐	☐	☐	☐	☐
High risk foot patients are reviewed by a hospital podiatrist	☐	☐	☐	☐	☐	☐
Active foot disease patients are seen by a hospital podiatrist within 24hours/next working day	☐	☐	☐	☐	☐	☐

Service Overview (Community)

* 63. What tool do you use to screen for diabetes foot disease?

- National Diabetes Foot Screening Tool
- I do not use a specific screening tool
- Locally developed tool
- I do not provide diabetic foot screening services
- Other (please name)

* 64. On average, how many patients with diabetes do you see per week?

* 65. Of your clients who are people with diabetes, how many appointments each week are DNA or cancelled appointments?

* 66. How did you answer the above question?

- Actual data
- Estimation
- Actual data and estimation

* 67. Do you provide out-of-hours diabetes consultations?

- Yes
- No

68. If yes, when are the out-of-hours sessions held in your area? (Tick all that apply)

- At weekends
- In the evenings
- Other (please specify)

* 69. Do you provide a drop-in service for patients?

- Yes
- No

* 70. Are you currently collecting data about the podiatry service?

- Yes
- No

71. If yes, what information is being collected?

* 72. Is this data shared?

- Yes
- No
- I do not collect podiatry service data

73. If yes, who is collected data shared with?

* 74. Has your department developed PPGs (procedures, pathways, policies and guidelines)?

Yes No

75. If yes, have the PPPGs developed been validated?

Yes No

76. We value your insight into diabetes care. Please use the space provided to describe your experiences of implementing the National Model of Diabetic Foot Care. If applicable, what are the changes you would make to the National Clinical Programme for Diabetes?

Providing Diabetes Foot Care (Podiatrists in Private Practice)

* 77. What percentage (%) of your client load are people with diabetes?

* 78. Are you aware of the National Model of Diabetic Foot Care?

- Yes
- No

* 79. In relation to diabetic foot disease, what percentage (%) of your clients fit the following categories:

Low risk

Moderate risk

High risk

Active Foot Disease

* 80. How did you answer the above question?

- Actual data
- Estimation
- Actual data and estimation

* 81. Please indicate the percentage (%) of clients referred to you from the following sources (from January 2017):

Self-referral

GP/Practice Nurse

Public Health Nurse

Diabetes Nurse Specialist

Community Podiatrist

Hospital Podiatrist

Hospital Consultant

* 82. How did you answer the above question?

- Actual data
- Estimation
- Actual data and estimation

* 83. In general, during your typical working week, do you carry out the following activities?

	Yes	No
Treat non-diabetic foot pathology such as nail conditions, corns, callous or verrucae	☐	☐
Diabetic foot care education	☐	☐
Refer high risk patients to hospital podiatrist	☐	☐
Treat high risk patients	☐	☐
Refer patients with active foot disease to the hospital podiatrist	☐	☐
Treat patients with active foot disease	☐	☐

Other (please specify)

84. If you answered 'No' to any of the statements above, please indicate your reasoning for this?

Treat non-diabetic foot pathology such as nail conditions, corns, callous or verrucae

Diabetic foot care education

Refer high risk patients to hospital podiatrist

Treat high risk patients

Refer patients with active foot disease to the hospital podiatrist

Treat patients with active foot disease

Service Overview (Private Practice)

* 85. What tool do you use to screen for diabetes foot disease?

- ◯ National Diabetes Foot Screening Tool
- ◯ Locally developed tool
- ◯ Other (please name)
- ◯ I do not use a specific screening tool
- ◯ I do not provide diabetic foot screening services

* 86. Has the private practice you work in developed PPGs (procedures, pathways, policies and guidelines)?

◯ Yes ◯ No

87. If yes, have the PPPGs developed been validated?

◯ Yes ◯ No

88. Please use the space provided to describe your experiences of treating people with diabetes as a private practitioner. Has anything changed since the National Model of Diabetic Foot Care was introduced?

Hospital & Community: Employment Details

* 89. Who are you employed and funded by?

	Employed by	Funded by
HSE	☐	☐
Community Hospital	☐	☐
Voluntary hospital	☐	☐
Local Diabetes Initiative	☐	☐
Diabetes Ireland	☐	☐
Other	☐	☐

Other (please specify)

* 90. In what community location(s) do you work? (Tick all that apply)

☐ Primary Care Centre

☐ Sole GP Practice

☐ Other (please specify)

* 91. What is the whole time equivalent (WTE) of your position? (e.g. 0.5, 0.75, 1)

In hospital

In the community

* 92. Please indicate the hospital model:

○ Model 2 ○ Model 3 ○ Model 4 ○ Don't know

* 93. What percentage (%) of your client load are people with diabetes?

In hospital

In the community

* 94. How did you answer the above question?

○ Actual data

○ Estimation

○ Actual data and estimation

Hospital & Community: Providing Diabetes Foot Care (Hospital)

* 95. Who are the multidisciplinary team members for diabetic foot care in your workplace?

- [ ] Dietitian
- [ ] Occupational Therapist
- [ ] Orthotist
- [ ] Physiotherapist
- [ ] Public Health Nurse
- [ ] Endocrinologist
- [ ] Psychologist
- [ ] Vascular Team
- [ ] Diabetes Nurse Specialist
- [ ] Other (please specify)

* 96. On average, what percentage (%) of your clients fit the following categories:

Moderate Risk

High Risk

Active Foot Disease

* 97. How did you answer the above question?

- ( ) Actual data
- ( ) Estimation
- ( ) Actual data and estimation

* 98. Please indicate the percentage (%) of clients referred to you from the following sources (from January 2017):

Self-referral

GP/Practice Nurse

Public Health Nurse

Diabetes Nurse Specialist

Community Podiatrist

Emergency Department

Internal (hospital) referral

* 99. How did you answer the above question?

- ( ) Actual data
- ( ) Estimation
- ( ) Actual data and estimation

* 100. Please indicate the percentage (%) of inappropriate referrals you have received in the last month: *(Inappropriate referrals are those which fall outside of your treatment responsibilities, according to the National Footcare Model and/or your job specification)*

* 101. How did you answer the above question?

- ( ) Actual data
- ( ) Estimation
- ( ) Actual data and estimation

102. If you receive inappropriate referrals, please indicate the main reasons for this:

* 103. What is the waiting period for patients accessing your service under the following categories:

Active Foot Disease

High Risk

Moderate Risk

Low Risk

* 104. How did you answer the above question?

- Actual data
- Estimation
- Actual data and estimation

* 105. Do you have referral access to the following services?

	I have referral access where I work	I have referral access to another hospital	I do not have referral access
Diabetes Nurse Specialist	☐	☐	☐
Endocrinologist/Diabetologist	☐	☐	☐
Vascular Surgery	☐	☐	☐
Orthopaedic Surgery	☐	☐	☐
Orthotists	☐	☐	☐

* 106. Do you have referral access to the following services:

	Yes	No
Chiropodist	☐	☐
GP/Practice Nurse	☐	☐
Community Podiatrist (Public)	☐	☐
Community Podiatrist (Private)	☐	☐
Public Health Nurse	☐	☐

Other (please specify)

* 107. In general, during your typical working week, do you carry out the following activities?

	Yes	No
Treat non-diabetic foot pathology such as nail conditions, corns, callous or verrucae	☐	☐
Refer patients with non-diabetic foot pathology such as nail conditions, corns, verrucae to the community podiatrist/chiropodist	☐	☐
Annual review of moderate risk patients	☐	☐
Annual review of high risk patients	☐	☐
Treat high risk patients	☐	☐
Provide a rapid access service (i.e. see referrals for active foot disease within 24 hours)	☐	☐
Treat patients with active foot disease	☐	☐
Weekly review to patients with active foot disease until the foot is healed	☐	☐
Educate the patient in diabetes related foot care	☐	☐
Record activity statistics	☐	☐

Other (please specify)

[ ]

108. If you answered 'No' to any of the following statements, please indicate the reason(s) for this:

Treat non-diabetic foot pathology such as nail conditions, corn, callous, or verrucae	
Refer patients with non-diabetic foot pathology to the community chiropodist/podiatrist	
Annual review of moderate risk patients	
Annual review of high risk patients	
Treat high risk patients	
Provide a rapid access service (i.e. see referrals for active foot disease within 24 hours)	
Treat patients with active foot disease	
Weekly review to patients with active foot disease until the foot is healed	
Educate the patient in diabetes related foot care	
Record activity statistics	

* 109. Please indicate your satisfaction with community services in your locality:

	Very satisfied	Satisfied	Neither satisfied nor unsatisfied	Unsatisfied	Very unsatisfied	I don't know if this happens
Appropriate screening is conducted by GPs/Practice Nurse	☐	☐	☐	☐	☐	☐
Patients at low risk of diabetic foot disease are managed in a GP practice	☐	☐	☐	☐	☐	☐
Appropriate referral pathways are used by GPs/Practice Nurses	☐	☐	☐	☐	☐	☐
Appropriate screening is conducted by podiatrists working in the community	☐	☐	☐	☐	☐	☐
Appropriate referral pathways are used by podiatrists working in the community	☐	☐	☐	☐	☐	☐
Annual review of moderate risk patients is completed by the community podiatrist	☐	☐	☐	☐	☐	☐

Service Overview (Hospital)

* 110. What tool do you use to screen for diabetes foot disease?

- National Diabetes Foot Screening Tool
- I do not use a specific screening tool
- Locally developed tool
- I do not provide diabetic foot screening services
- Other (please name)

* 111. On average, how many patients with diabetes do you see per week?

* 112. Of your clients who are people with diabetes, how many appointments each week are DNA or cancelled appointments?

* 113. How did you answer the above question?

- Actual data
- Estimation
- Actual data and estimation

* 114. Do you provide out-of-hours diabetes consultations?

- Yes
- No

115. If yes, when are the out-of-hours sessions held in your area? (Tick all that apply)

- At weekends
- In the evenings
- Other (please specify)

* 116. Do you provide a drop-in service for patients?

- Yes
- No

* 117. Are you currently collecting data about the podiatry service?

- Yes
- No

118. If yes, what information is being collected?

* 119. Is this data shared?

- Yes
- No
- I do not collect podiatry service data

120. If yes, who is collected data shared with?

* 121. Has your department developed PPGs (procedures, pathways, policies and guidelines)?

Yes No

122. If yes, have the PPPGs developed been validated?

Yes No

123. We value your insight into diabetes care. Please use the space provided to describe your experiences of implementing the National Model of Diabetic Foot Care. If applicable, what are the changes you would make to the National Clinical Programme for Diabetes?

Podiatrists working in hospitals and in the community

We are grateful that you have completed a series of questions relating to your work as a hospital podiatrist. We would appreciate if you would spend a few minutes answering a similar set of questions in about your role as a podiatrist working in community settings. Thank you for your time.

Hospital & Community: Providing Diabetes Foot Care (Community)

* 124. Who are the established multidisciplinary team (MDT) members for diabetic foot care in your workplace?

- [ ] Dietitian
- [ ] Diabetes Nurse Specialist
- [ ] Public Health Nurse
- [ ] Practice Nurse
- [ ] GP
- [ ] Other (please specify)

* 125. In relation to diabetic foot disease, what percentage (%) of your clients fit the following categories:

Low risk

Moderate risk

High risk

Active Foot Disease

* 126. How did you answer the above question?

- ( ) Actual data
- ( ) Estimation
- ( ) Actual data and estimation

* 127. Please indicate the percentage (%) of clients referred to you from the following sources (since January 2017):

Self-referral

GP/Practice Nurse

Public Health Nurse

Diabetes Nurse Specialist

Hospital Podiatrist

Hospital Consultant

* 128. How did you answer the above question?

- ( ) Actual data
- ( ) Estimation
- ( ) Actual data and estimation

* 129. Please indicate the percentage (%) of inappropriate referrals you have received in the last month:*(Inappropriate referrals are those which fall outside of your treatment responsibilities, according to the National Footcare Model and/or your job specification)*

* 130. How did you answer the above question?

- ( ) Actual data
- ( ) Estimation
- ( ) Actual data and estimation

131. If you receive inappropriate referrals, please indicate the main reasons for this:

* 132. What is the waiting period for patients accessing your service under the following categories:

Active Foot Disease

High Risk

Moderate Risk

Low Risk

* 133. How did you answer the above question?

- Actual data
- Estimation
- Actual data and estimation

* 134. Do you have referral access to the following services:

	Yes	No
Chiropodist		
GP/Practice Nurse		
Hospital Podiatrist		
Diabetes Nurse Specialist		
Public Health Nurse		
Podiatrist (Private)		

Other (please specify)

* 135. In general, during your typical working week, do you carry out the following activities?

	Yes	No
Treat non-diabetic foot pathology such as nail conditions, corns, callous or verrucae	☐	☐
Diabetic foot care education	☐	☐
Screen and categorise risk of diabetic foot disease (i.e. low / mod / high)	☐	☐
Annual review of low risk patients	☐	☐
Annual review of moderate risk patients	☐	☐
Review high risk patients	☐	☐
Refer high risk patients to the hospital podiatrist	☐	☐
Refer patients with active foot disease to the hospital podiatrist	☐	☐
Record activity statistics	☐	☐

Other (please specify)

____

136. If you answered 'No' to any of the following statements, please indicate the reason(s) for this:

Treat non-diabetic foot pathology such as nail conditions, corn, callous, or verrucae ____________________

Diabetic foot care education ____________________

Screen and categorise risk of diabetic foot disease (i.e. low / mod / high) ____________________

Annual review of low risk patients ____________________

Annual review of moderate risk patients ____________________

Review high risk patients ____________________

Refer high risk patients to the hospital podiatrist ____________________

Refer patients with active foot disease to the hospital podiatrist ____________________

Record activity statistics ____________________

* 137. Please indicate your satisfaction with local podiatry services:

	Very satisfied	Satisfied	Neither satisfied nor unsatisfied	Unsatisfied	Very unsatisfied	I don't know if this happens
Appropriate screening is conducted by GPs/Practice Nurse	☐	☐	☐	☐	☐	☐
Practice Nurse uses the national screening tool	☐	☐	☐	☐	☐	☐
Patients at low risk of diabetic foot disease are managed in a GP Practice	☐	☐	☐	☐	☐	☐
Public health nurses use the national screening tool	☐	☐	☐	☐	☐	☐
High risk foot patients are reviewed by a hospital podiatrist	☐	☐	☐	☐	☐	☐
Active foot disease patients are seen by a hospital podiatrist within 24hours/next working day	☐	☐	☐	☐	☐	☐

Service Overview (Community)

* 138. What tool do you use to screen for diabetes foot disease?

- ☐ National Diabetes Foot Screening Tool
- ☐ I do not use a specific screening tool
- ☐ Locally developed tool
- ☐ I do not provide diabetic foot screening services
- ☐ Other (please name)

* 139. On average, how many patients with diabetes do you see per week?

* 140. Of your clients who are people with diabetes, how many appointments each week are DNA or cancelled appointments?

* 141. How did you answer the above question?

- ◯ Actual data
- ◯ Estimation
- ◯ Actual data and estimation

* 142. Do you provide out-of-hours diabetes consultations?

◯ Yes ◯ No

143. If yes, when are the out-of-hours sessions held in your area? (Tick all that apply)

- ☐ At weekends
- ☐ In the evenings
- ☐ Other (please specify)

* 144. Do you provide a drop-in service for patients?

◯ Yes ◯ No

* 145. Are you currently collecting data about the podiatry service?

◯ Yes ◯ No

* 146. If yes, what information is being collected?

* 147. Is this data shared?

◯ Yes ◯ No ◯ I do not collect podiatry service data

148. If yes, who is collected data shared with?

* 149. Has your department developed PPGs (procedures, pathways, policies and guidelines)?

Yes No

150. If yes, have the PPPGs developed been validated?

Yes No

151. We value your insight into diabetes care. Please use the space provided to describe your experiences of implementing the National Model of Diabetic Foot Care. If applicable, what are the changes you would make to the National Clinical Programme for Diabetes?

Hospital & Private Practice: Employment Details (Hospital)

* 152. Who are you employed and funded by?

	Employed by	Funded by
HSE	☐	☐
Community Hospital	☐	☐
Voluntary hospital	☐	☐
Self	☐	☐
Other Individual/Company	☐	☐
Diabetes Ireland	☐	☐
Local Diabetes Initiative	☐	☐
Other	☐	☐

Other (please specify)

* 153. What is the whole time equivalent (WTE) of your position? (e.g. 0.5, 0.75, 1)

In the hospital

In private practice

* 154. Please indicate the hospital model:

○ Model 2 ○ Model 3 ○ Model 4 ○ Don't know

* 155. What percentage (%) of your client load are people with diabetes?

In hospital

In private practice

* 156. How did you answer the above question?

○ Actual data ○ Estimation ○ Actual data and estimation

Hospital & Private Practice: Providing Diabetes Foot Care (Hospital)

* 157. Who are the multidisciplinary team members for diabetic foot care in your workplace?

- [ ] Dietitian
- [ ] Occupational Therapist
- [ ] Orthotist
- [ ] Physiotherapist
- [ ] Public Health Nurse
- [ ] Endocrinologist
- [ ] Psychologist
- [ ] Vascular Team
- [ ] Diabetes Nurse Specialist
- [ ] Other (please specify)

* 158. Please indicate the percentage (%) of clients referred to you from the following sources (from January 2017):

Self-referral

GP/Practice Nurse

Public Health Nurse

Diabetes Nurse Specialist

Community Podiatrist

Emergency Department

Internal (hospital) referral

* 159. How did you answer the above question?

- ( ) Actual data
- ( ) Estimation
- ( ) Actual data and estimation

* 160. Please indicate the percentage (%) of inappropriate referrals you have received in the last month: *(Inappropriate referrals are those which fall outside of your treatment responsibilities, according to the National Footcare Model and/or your job specification)*

* 161. How did you answer the above question?

- ( ) Actual data
- ( ) Estimation
- ( ) Actual data and estimation

162. If you receive inappropriate referrals, please indicate the main reasons for this:

* 163. Do you have referral access to the following services?

	I have referral access where I work	I have referral access to another hospital	I do not have referral access
Diabetes Nurse Specialist	☐	☐	☐
Endocrinologist/Diabetologist	☐	☐	☐
Vascular Surgery	☐	☐	☐
Orthopaedic Surgery	☐	☐	☐
Orthotists	☐	☐	☐

* 164. Do you have referral access to the following services in the community:

	Yes	No
Chiropodist	☐	☐
GP/Practice Nurse	☐	☐
Community Podiatrist (Public)	☐	☐
Community Podiatrist (Private)	☐	☐
Public Health Nurse	☐	☐

Other (please specify)

* 165. On average, what percentage (%) of your clients are:

Moderate Risk

High Risk

Active Foot Disease

* 166. How did you answer the above question?

○ Actual data ○ Estimation ○ Actual data and estimation

* 167. What is the waiting period for patients accessing your service under the following categories:

Active Foot Disease

High Risk

Moderate Risk

Low Risk

* 168. How did you answer the above question?

○ Actual data ○ Estimation ○ Actual data and estimation

169. Briefly indicate the main reasons for this waiting list period?

* 170. In general, during your typical working week, do you carry out the following activities?

	Yes	No
Treat non-diabetic foot pathology such as nail conditions, corns, callous or verrucae	☐	☐
Refer patients with non-diabetic foot pathology such as nail conditions, corns, verrucae to the community podiatrist/chiropodist	☐	☐
Annual review of moderate risk patients	☐	☐
Annual review of high risk patients	☐	☐
Treat high risk patients	☐	☐
Provide a rapid access service (i.e. see referrals for active foot disease within 24 hours)	☐	☐
Treat patients with active foot disease	☐	☐
Weekly review to patients with active foot disease until the foot is healed	☐	☐
Educate the patient in diabetes related foot care	☐	☐
Record activity statistics	☐	☐

Other (please specify)

171. If you answered 'No' to any of the following statements, please indicate the reason(s) for this:

Treat non-diabetic foot pathology such as nail conditions, corn, callous, or verrucae	
Refer patients with non-diabetic foot pathology to the community chiropodist/podiatrist	
Annual review of moderate risk patients	
Annual review of high risk patients	
Treat high risk patients	
Provide a rapid access service (i.e. see referrals for active foot disease within 24 hours)	
Treat patients with active foot disease	
Weekly review to patients with active foot disease until the foot is healed	
Educate the patient in diabetes related foot care	
Record activity statistics	

* 172. Please indicate your satisfaction with community services in your locality:

	Very satisfied	Satisfied	Neither satisfied nor unsatisfied	Unsatisfied	Very unsatisfied	I don't know if this happens
Appropriate screening is conducted by GPs/Practice Nurse	☐	☐	☐	☐	☐	☐
Patients at low risk of diabetic foot disease are managed in a GP practice	☐	☐	☐	☐	☐	☐
Appropriate referral pathways are used by GPs/Practice Nurses	☐	☐	☐	☐	☐	☐
Appropriate screening is conducted by podiatrists working in the community	☐	☐	☐	☐	☐	☐
Appropriate referral pathways are used by podiatrists working in the community	☐	☐	☐	☐	☐	☐
Annual review of moderate risk patients is completed by the community podiatrist	☐	☐	☐	☐	☐	☐

Service Overview (Hospital)

* 173. What tool do you use to screen for diabetes foot disease?

- National Diabetes Foot Screening Tool
- I do not use a specific screening tool
- Locally developed tool
- I do not provide diabetic foot screening services
- Other (please name)

* 174. On average, how many patients with diabetes do you see per week?

* 175. Of your clients who are people with diabetes, how many appointments each week are DNA or cancelled appointments?

* 176. How did you answer the above question?

- Actual data
- Estimation
- Actual data and estimation

* 177. Do you provide out-of-hours diabetes consultations?

- Yes
- No

178. If yes, when are the out-of-hours sessions held in your area? (Tick all that apply)

- At weekends
- In the evenings
- Other (please specify)

* 179. Do you provide a drop-in service for patients?

- Yes
- No

* 180. Are you currently collecting data about the podiatry service?

- Yes
- No

181. If yes, what information is being collected?

* 182. Is this data shared?

- Yes
- No
- I do not collect data

183. If yes, who is collected data shared with?

* 184. Has your department developed PPGs (procedures, pathways, policies and guidelines)?

Yes No

185. If yes, have the PPPGs developed been validated?

Yes No

186. We value your insight into diabetes care. Please use the space provided to describe your experiences of implementing the National Model of Diabetic Foot Care. If applicable, what are the changes you would make to the National Clinical Programme for Diabetes?

Podiatrists working in hospitals and in private practice

We are grateful that you have completed a series of questions relating to your work as a hospital podiatrist. We would appreciate if you would spend a few minutes answering a similar set of questions in relation to your role as a podiatrist working in private practice. Thank you for your time.

Hospital & Private Practice: Providing Diabetes Foot Care (Podiatrists in Private Practice)

* 187. In relation to diabetic foot disease, what percentage (%) of your clients fit the following categories:

Low risk ________

Moderate risk ________

High risk ________

Active Foot Disease ________

* 188. How did you answer the above question?

- ○ Actual data
- ○ Estimation
- ○ Actual data and estimation

* 189. Please indicate the percentage (%) of clients referred to you from the following sources (from January 2017):

Self-referral ________

GP/Practice Nurse ________

Public Health Nurse ________

Diabetes Nurse Specialist ________

Community Podiatrist ________

Hospital Podiatrist ________

Hospital Consultant ________

* 190. How did you answer the above question?

- ○ Actual data
- ○ Estimation
- ○ Actual data and estimation

* 191. In general, during your typical working week, do you carry out the following activities?

	Yes	No
Treat non-diabetic foot pathology such as nail conditions, corns, callous or verrucae	☐	☐
Diabetic foot care education	☐	☐
Refer high risk patients to hospital podiatrist	☐	☐
Treat high risk patients	☐	☐
Refer patients with active foot disease to the hospital podiatrist	☐	☐
Treat patients with active foot disease	☐	☐

Other (please specify)

192. If you answered 'No' to any of the statements above, please indicate your reasoning for this?

Treat non-diabetic foot pathology such as nail conditions, corns, callous or verrucae

Diabetic foot care education

Refer high risk patients to hospital podiatrist

Treat high risk patients

Refer patients with active foot disease to the hospital podiatrist

Treat patients with active foot disease

Service Overview (Private Practice)

* 193. What tool do you use to screen for diabetes foot disease?

- ◯ National Diabetes Foot Screening Tool
- ◯ I do not use a specific screening tool
- ◯ Locally developed tool
- ◯ I do not provide diabetic foot screening services
- ◯ Other (please name)

* 194. Has the private practice you work in developed PPGs (procedures, pathways, policies and guidelines)?

◯ Yes ◯ No

195. If yes, have the PPPGs developed been validated?

◯ Yes ◯ No

196. Please use the space provided to describe your experiences of treating people with diabetes as a private practitioner. Has anything changed since the National Model of Diabetic Foot Care was introduced?

Community & Private Practice: Employment Details (Community)

* 197. Who are you employed and funded by?

	Employed by	Funded by
HSE	☐	☐
Diabetes Ireland	☐	☐
Local Diabetes Initiative	☐	☐
Self	☐	☐
Other Company/Business owner	☐	☐
Other	☐	☐

Other (please specify)

* 198. What is the whole time equivalent (WTE) of your position? (e.g. 0.5, 0.75, 1)

In the community

In private practice

* 199. In what location(s) do you work? (Tick all that apply)

- ☐ Primary Care Centre
- ☐ Sole GP Practice
- ☐ Privately owned clinic
- ☐ Other (please specify)

* 200. Are you aware of the National Model of Diabetic Foot Care?

- ○ Yes
- ○ No

* 201. What percentage (%) of your client load are people with diabetes?

In the community

In private prctice

Community & Private Practice: Providing Diabetes Foot Care (Community)

* 202. Who are the established multidisciplinary team (MDT) members for diabetic foot care in your workplace?

- [ ] Dietitian
- [ ] Diabetes Nurse Specialist
- [ ] Public Health Nurse
- [ ] Practice Nurse
- [ ] GP
- [ ] Other (please specify)

* 203. Please indicate the percentage (%) of clients referred to you from the following sources (since January 2017):

Self-referral

GP/Practice Nurse

Public Health Nurse

Diabetes Nurse Specialist

Hospital Podiatrist

Hospital Consultant

* 204. How did you answer the above question?

- ( ) Actual data
- ( ) Estimation
- ( ) Actual data and estimation

* 205. In relation to diabetic foot disease, what percentage (%) of your clients fit the following categories:

Low risk

Moderate risk

High risk

Active Foot Disease

* 206. How did you answer the above question?

- ( ) Actual data
- ( ) Estimation
- ( ) Actual data and estimation

* 207. Please indicate the percentage (%) of inappropriate referrals you have received in the last month:*(Inappropriate referrals are those which fall outside of your treatment responsibilities, according to the National Footcare Model and/or your job specification)*

* 208. How did you answer the above question?

- ( ) Actual data
- ( ) Estimation
- ( ) Actual data and estimation

209. If you receive inappropriate referrals, please indicate the main reasons for this:

* 210. What is the waiting period for patients accessing your service under the following categories:

Active Foot Disease

High Risk

Moderate Risk

Low Risk

* 211. How did you answer the above question?

- Actual data
- Estimation
- Actual data and estimation

* 212. Do you have referral access to the following services:

	Yes	No
Chiropodist		
GP/Practice Nurse		
Hospital Podiatrist		
Diabetes Nurse Specialist		
Podiatrist (Private)		
Public Health Nurse		

Other (please specify)

* 213. In general, during your typical working week, do you carry out the following activities?

	Yes	No
Treat non-diabetic foot pathology such as nail conditions, corns, callous or verrucae	☐	☐
Diabetic foot care education	☐	☐
Screen and categorise risk of diabetic foot disease (i.e. low / mod / high)	☐	☐
Annual review of low risk patients	☐	☐
Annual review of moderate risk patients	☐	☐
Review high risk patients	☐	☐
Refer high risk patients to the hospital podiatrist	☐	☐
Refer patients with active foot disease to the hospital podiatrist	☐	☐
Record activity statistics	☐	☐

Other (please specify)

214. If you answered 'No' to any of the following statements, please indicate the reason(s) for this:

Treat non-diabetic foot pathology such as nail conditions, corn, callous, or verrucae

Diabetic foot care education

Screen and categorise risk of diabetic foot disease (i.e. low / mod / high)

Annual review of low risk patients

Annual review of moderate risk patients

Review high risk patients

Refer high risk patients to the hospital podiatrist

Refer patients with active foot disease to the hospital podiatrist

Record activity statistics

* 215. Please indicate your satisfaction with local podiatry services:

	Very satisfied	Satisfied	Neither satisfied nor unsatisfied	Unsatisfied	Very unsatisfied	I don't know if this happens
Appropriate screening is conducted by GPs/Practice Nurse	☐	☐	☐	☐	☐	☐
Practice Nurse uses the national screening tool	☐	☐	☐	☐	☐	☐
Patients at low risk of diabetic foot disease are managed in a GP Practice	☐	☐	☐	☐	☐	☐
Public health nurses use the national screening tool	☐	☐	☐	☐	☐	☐
High risk foot patients are reviewed by a hospital podiatrist	☐	☐	☐	☐	☐	☐
Active foot disease patients are seen by a hospital podiatrist within 24hours/next working day	☐	☐	☐	☐	☐	☐

Service Overview (Community)

* 216. What tool do you use to screen for diabetes foot disease?

- ◯ National Diabetes Foot Screening Tool
- ◯ I do not use a specific screening tool
- ◯ Locally developed tool
- ◯ I do not provide diabetic foot screening services
- ◯ Other (please name)

* 217. On average, how many patients with diabetes do you see per week?

* 218. Of your clients who are people with diabetes, how many appointments each week are DNA or cancelled appointments?

* 219. How did you answer the above question?

- ◯ Actual data
- ◯ Estimation
- ◯ Actual data and estimation

* 220. Do you provide out-of-hours diabetes consultations?

- ◯ Yes
- ◯ No

221. If yes, when are the out-of-hours sessions held in your area? (Tick all that apply)

- ☐ At weekends
- ☐ In the evenings
- ☐ Other (please specify)

* 222. Do you provide a drop-in service for patients?

- ◯ Yes
- ◯ No

* 223. Are you currently collecting data about the podiatry service?

- ◯ Yes
- ◯ No

224. If yes, what information is being collected?

* 225. Is this data shared?

- ◯ Yes
- ◯ No
- ◯ I do not collect podiatry service data

226. If yes, who is collected data shared with?

* 227. Has your department developed PPGs (procedures, pathways, policies and guidelines)?

Yes No

228. If yes, have the PPPGs developed been validated?

Yes No

229. We value your insight into diabetes care. Please use the space provided to describe your experiences of implementing the National Model of Diabetic Foot Care. If applicable, what are the changes you would make to the National Clinical Programme for Diabetes?

Podiatrists working in the community and in private practice

We are grateful that you have completed a series of questions relating to your work as a community podiatrist. We would appreciate if you would spend a few minutes answering a similar set of questions in relation to your role as a podiatrist working in private practice. Thank you for your time.

Community & Private Practice: Providing Diabetes Foot Care (Podiatrists in Private Practice)

* 230. In relation to diabetic foot disease, what percentage (%) of your clients fit the following categories:

Low risk

Moderate risk

High risk

Active Foot Disease

* 231. How did you answer the above question?

○ Actual data ○ Estimation ○ Actual data and estimation

* 232. Please indicate the percentage (%) of clients referred to you from the following sources (from January 2017):

Self-referral

GP/Practice Nurse

Public Health Nurse

Diabetes Nurse Specialist

Community Podiatrist

Hospital Podiatrist

Hospital Consultant

* 233. How did you answer the above question?

○ Actual data ○ Estimation ○ Actual data and estimation

* 234. In general, during your typical working week, do you carry out the following activities?

	Yes	No
Treat non-diabetic foot pathology such as nail conditions, corns, callous or verrucae	☐	☐
Diabetic foot care education	☐	☐
Refer high risk patients to hospital podiatrist	☐	☐
Treat high risk patients	☐	☐
Refer patients with active foot disease to the hospital podiatrist	☐	☐
Treat patients with active foot disease	☐	☐

Other (please specify)

235. If you answered 'No' to any of the statements above, please indicate your reasoning for this?

Treat non-diabetic foot pathology such as nail conditions, corns, callous or verrucae

Diabetic foot care education

Refer high risk patients to hospital podiatrist

Treat high risk patients

Refer patients with active foot disease to the hospital podiatrist

Treat patients with active foot disease

Service Overview (Private Practice)

* 236. What tool do you use to screen for diabetes foot disease?

- ○ National Diabetes Foot Screening Tool
- ○ Locally developed tool
- ○ Other (please name)
- ○ I do not use a specific screening tool
- ○ I do not provide diabetic foot screening services

* 237. Has the private practice you work in developed PPGs (procedures, pathways, policies and guidelines)?

○ Yes ○ No

238. If yes, have the PPPGs developed been validated?

○ Yes ○ No

239. Please use the space provided to describe your experiences of treating people with diabetes as a private practitioner. Has anything changed since the National Model of Diabetic Foot Care was introduced?

Education & Continued Professional Development

* 240. Please describe your continuing professional development activities over the past 12 months:

- [ ] Often undertake/attend CPD activities
- [ ] Rarely undertake/attend CPD activities
- [ ] Sometimes undertake/attend CPD activities
- [ ] Never undertake/attend CPD activities

* 241. Is there a protected budget for continuing professional development?

- ( ) Yes
- ( ) No

* 242. Do you have protected time for continuing professional development?

- ( ) Yes
- ( ) No

* 243. Please indicate the continuing professional development activities you engage in:

- [ ] Conference attendance
- [ ] Education/upskilling (educational course)
- [ ] Attend National Days for CPD (facilitated by the NCPD)
- [ ] Education/upskilling (workshop)
- [ ] Attend National Days for CPD (not facilitated by the NCPD)
- [ ] None
- [ ] Educate other professionals about podiatry
- [ ] Other (please specify)

* 244. Do you practice self-directed CPD activities?

- [ ] Review journal articles/literature
- [ ] Reflective practice
- [ ] Watch webinars
- [ ] I do not engage in these activities
- [ ] Undertake learning on HSELand
- [ ] Other (please specify)

* 245. Do you provide a structured education programme to any of the following professional groups?

- [ ] GP
- [ ] Allied health professionals
- [ ] Practice Nurse
- [ ] Medical staff in nursing homes
- [ ] Nursing staff in hospitals
- [ ] I do not provide structured education to other professional groups
- [ ] Medical staff in hospitals
- [ ] Other (please specify)

246. If you provide structured education, how is the education provided? (Tick all that apply)

- [ ] Information only
- [ ] One-on-one sessions
- [ ] Group sessions
- [ ] Other (please specify)

* 247. Are you aware of the CORU registration plan for podiatrists?

- ( ) Yes
- ( ) No

* 248. Have you prepared an appropriate and up-to-date CPD portfolio for CORU?

- ( ) Yes
- ( ) No

End of survey

Thank you for taking the time to complete the National Survey of Podiatrists. Your contribution will provide valuable understanding of the organisation of podiatry services in Ireland and is greatly appreciated by the research team. If you require further information or wish to withdraw your information from the study, please email ESPRIT at esprit@ucc.ie
